# Using “Omics” and Integrated Multi-Omics Approaches to Guide Probiotic Selection to Mitigate Chytridiomycosis and Other Emerging Infectious Diseases

**DOI:** 10.3389/fmicb.2016.00068

**Published:** 2016-02-02

**Authors:** Eria A. Rebollar, Rachael E. Antwis, Matthew H. Becker, Lisa K. Belden, Molly C. Bletz, Robert M. Brucker, Xavier A. Harrison, Myra C. Hughey, Jordan G. Kueneman, Andrew H. Loudon, Valerie McKenzie, Daniel Medina, Kevin P. C. Minbiole, Louise A. Rollins-Smith, Jenifer B. Walke, Sophie Weiss, Douglas C. Woodhams, Reid N. Harris

**Affiliations:** ^1^Department of Biology, James Madison UniversityHarrisonburg, VA, USA; ^2^Unit for Environmental Sciences and Management, North-West UniversityPotchefstroom, South Africa; ^3^Institute of Zoology, Zoological Society of LondonLondon, UK; ^4^School of Environment and Life Sciences, University of SalfordSalford, UK; ^5^Center for Conservation and Evolutionary Genetics, Smithsonian Conservation Biology Institute, National Zoological ParkWashington, DC, USA; ^6^Department of Biological Sciences, Virginia TechBlacksburg, VA, USA; ^7^Zoological Institute, Technische Universität BraunschweigBraunschweig, Germany; ^8^Rowland Institute, Harvard UniversityCambridge, MA, USA; ^9^Department of Ecology and Evolutionary Biology, University of ColoradoBoulder, CO, USA; ^10^Department of Zoology, Biodiversity Research Centre, University of British ColumbiaVancouver, BC, Canada; ^11^Department of Chemistry, Villanova UniversityVillanova, PA, USA; ^12^Department of Pathology, Microbiology and Immunology and Department of Pediatrics, Vanderbilt University School of Medicine, Department of Biological Sciences, Vanderbilt UniversityNashville, TN, USA; ^13^Department of Chemical and Biological Engineering, University of Colorado at BoulderBoulder, CO, USA; ^14^Department of Biology, University of Massachusetts BostonBoston, MA, USA

**Keywords:** probiotics, emerging diseases, metagenomics, transcriptomics, metabolomics, amphibians

## Abstract

Emerging infectious diseases in wildlife are responsible for massive population declines. In amphibians, chytridiomycosis caused by *Batrachochytrium dendrobatidis*, *Bd*, has severely affected many amphibian populations and species around the world. One promising management strategy is probiotic bioaugmentation of antifungal bacteria on amphibian skin. *In vivo* experimental trials using bioaugmentation strategies have had mixed results, and therefore a more informed strategy is needed to select successful probiotic candidates. Metagenomic, transcriptomic, and metabolomic methods, colloquially called “omics,” are approaches that can better inform probiotic selection and optimize selection protocols. The integration of multiple omic data using bioinformatic and statistical tools and *in silico* models that link bacterial community structure with bacterial defensive function can allow the identification of species involved in pathogen inhibition. We recommend using 16S rRNA gene amplicon sequencing and methods such as indicator species analysis, the Kolmogorov–Smirnov Measure, and co-occurrence networks to identify bacteria that are associated with pathogen resistance in field surveys and experimental trials. In addition to 16S amplicon sequencing, we recommend approaches that give insight into symbiont function such as shotgun metagenomics, metatranscriptomics, or metabolomics to maximize the probability of finding effective probiotic candidates, which can then be isolated in culture and tested in persistence and clinical trials. An effective mitigation strategy to ameliorate chytridiomycosis and other emerging infectious diseases is necessary; the advancement of omic methods and the integration of multiple omic data provide a promising avenue toward conservation of imperiled species.

## Introduction

Emerging infectious diseases (EIDs) in wildlife pose a grave threat to biodiversity (Wake and Vredenburg, 2008; Blehert et al., 2009; Fisher et al., 2009, 2012; Price et al., 2014; Schrope, 2014). Examples of EIDs caused by fungal pathogens include white-nose syndrome in bats (Blehert et al., 2009) and chytridiomycosis in amphibians (Berger et al., 1998). The latter, caused by *Batrachochytrium dendrobatidis* (*Bd*), is considered the greatest disease threat to biodiversity at the current time (Wake and Vredenburg, 2008). Recently, a newly described chytrid fungal species, *B. salamandrivorans* (*Bsal*) has been identified as the causal agent of chytridiomycosis in salamanders and is causing many salamander populations declines in Europe (Martel et al., 2013, 2014; Yap et al., 2015). Several strategies have been proposed to contend against EIDs in amphibians (Fisher et al., 2012; Woodhams et al., 2012; McMahon et al., 2014; Langwig et al., 2015) including vaccination, selective breeding and the use of probiotic bioaugmentation (Harris et al., 2006, 2009; Woodhams et al., 2007; Stice and Briggs, 2010; McMahon et al., 2014; Hoyt et al., 2015). Successful implementation of the later approach for the conservation of wild populations will benefit from further laboratory and field-testing, particularly when informed by integrated multi-omics methods.

There is growing evidence that probiotic therapy in particular could be a promising approach to mitigating disease in a variety of organisms, including human, plant crop, and wildlife systems (Harris et al., 2009; Sánchez et al., 2013; Akhter et al., 2015; Forster and Lawley, 2015; Hoyt et al., 2015; Papadimitriou et al., 2015). A relevant case comes from studies on plant-microbial interactions, which have identified several bacterial taxa involved in protection of plant crops against pathogens and extreme environmental stressors as well as in nutrient availability (Berlec, 2012). Some of these microorganisms have been widely and successfully used as bio-fertilizers or bio-controls in plant agriculture (Bhardwaj et al., 2014; Lakshmanan et al., 2014). In amphibians, the approach that is currently being developed is probiotic bioaugmentation, which is the establishment and augmentation of protective microbes that are already naturally occurring on at least some individuals in a population or community (Bletz et al., 2013). Bioaugmentation has prevented morbidity and mortality otherwise caused by *Bd* during laboratory-based and field-based trials for some amphibian species (Harris et al., 2006, 2009, Bletz et al., 2013). However, application of probiotics has been ineffective in other amphibian species (Becker et al., 2011, 2015a; Küng et al., 2014). The mixed success of probiotics could in part be caused by the selection of ineffective probiotic candidates because knowledge about the diversity of the microbiota and the ecological interactions occurring within these communities was lacking. For example, initial “training” of the immune system by early symbiotic colonists during development and priority effects of the microbial community, may exert strong influences on community resilience and colonization potential of probiotics (Reid et al., 2011; Hawkes and Keitt, 2015).

In an effort to improve the chances of a positive outcome from the use of amphibian probiotics, a protocol that filters out ineffective candidates has been proposed (Bletz et al., 2013). This method was designed to identify successful probiotics for disease mitigation and species survival based on culture-dependent data. In addition to the Bletz et al. (2013) filtering protocol, a mucosome assay, which aims to measure the protective function of the skin mucus, has recently been developed and applied to test potential probiotics (Woodhams et al., 2014).

As new technologies and methods are being developed, it is desirable to further improve the filtering protocol with additional methods that can be used to facilitate probiotic candidate selection to increase the likelihood of success. In particular, high-throughput molecular techniques, colloquially called “omics” methods, have greatly increased our ability to characterize the taxonomic and genetic structure of bacterial communities, to estimate their functional capabilities and to evaluate their responses to stressors or pathogens (Grice and Segre, 2011; Fierer et al., 2012; Greenblum et al., 2012; Knief et al., 2012; Jorth et al., 2014). Some of the omics methods developed to date are gene amplicon sequencing, shotgun metagenomics, transcriptomics, proteomics, and metabolomics. Several studies and extensive reviews on these high-throughput molecular methods can be found in the literature (Fiehn, 2002; Dettmer et al., 2007; Caporaso et al., 2011, 2012; Stewart et al., 2011; Altelaar et al., 2012; McGettigan, 2013; Franzosa et al., 2015; Loman and Pallen, 2015). Moreover, integrated multi-omics, which we define as the integrative analysis of data obtained from multiple omic methods, has the potential to greatly advance our understanding of ecological interactions occurring in microbial communities (Borenstein, 2012; McHardy et al., 2013; Meng et al., 2014). In this review, we establish an omics and integrated multi-omics framework with the aim of increasing the chances of selecting effective probiotic bacteria and achieving a successful disease mitigation strategy against EIDs. While these principles are applicable to other biological systems, for example in humans (Sánchez et al., 2013; Buffie et al., 2015; Forster and Lawley, 2015), we focus on applying these principles to the amphibian system, emphasizing current omics methods that have been explored in amphibians such as 16S rRNA gene amplicon sequencing (hereafter 16S amplicon sequencing), shotgun metagenomics, transcriptomics, and metabolomics. However, other omics methods such as proteomics could be relevant in future studies to understand the interactions between hosts, pathogens and host-associated microbial communities.

In this review, we will (1) provide relevant knowledge about the skin microbiome in amphibians; (2) proceed with a description of the omics and integrated multi-omics methods that have been or could be applied to the amphibian system; (3) describe how omics and integrated multi-omics approaches can be incorporated into a previously described filtering protocol to identify probiotic candidates (Bletz et al., 2013); (4) provide important considerations and future directions that are relevant to the success of probiotic selection supported by multi-omics data. It is important to note that the omics methods as well as the statistical, modeling and integrative methods mentioned in this review are a subset of the current methods available and additional methods can also be used to identify successful probiotic candidates.

## Ecology of the Amphibian Skin Microbiome

The amphibian skin microbiome is defined as the microbiota and its combined genetic material present on the skin. Determining the main drivers of the assembly of the skin microbiome through the use of omic methods and culture-dependent approaches may greatly enhance our ability to develop successful probiotic treatments and prevent amphibian population declines caused by chytridiomycosis.

**FIGURE 1:**
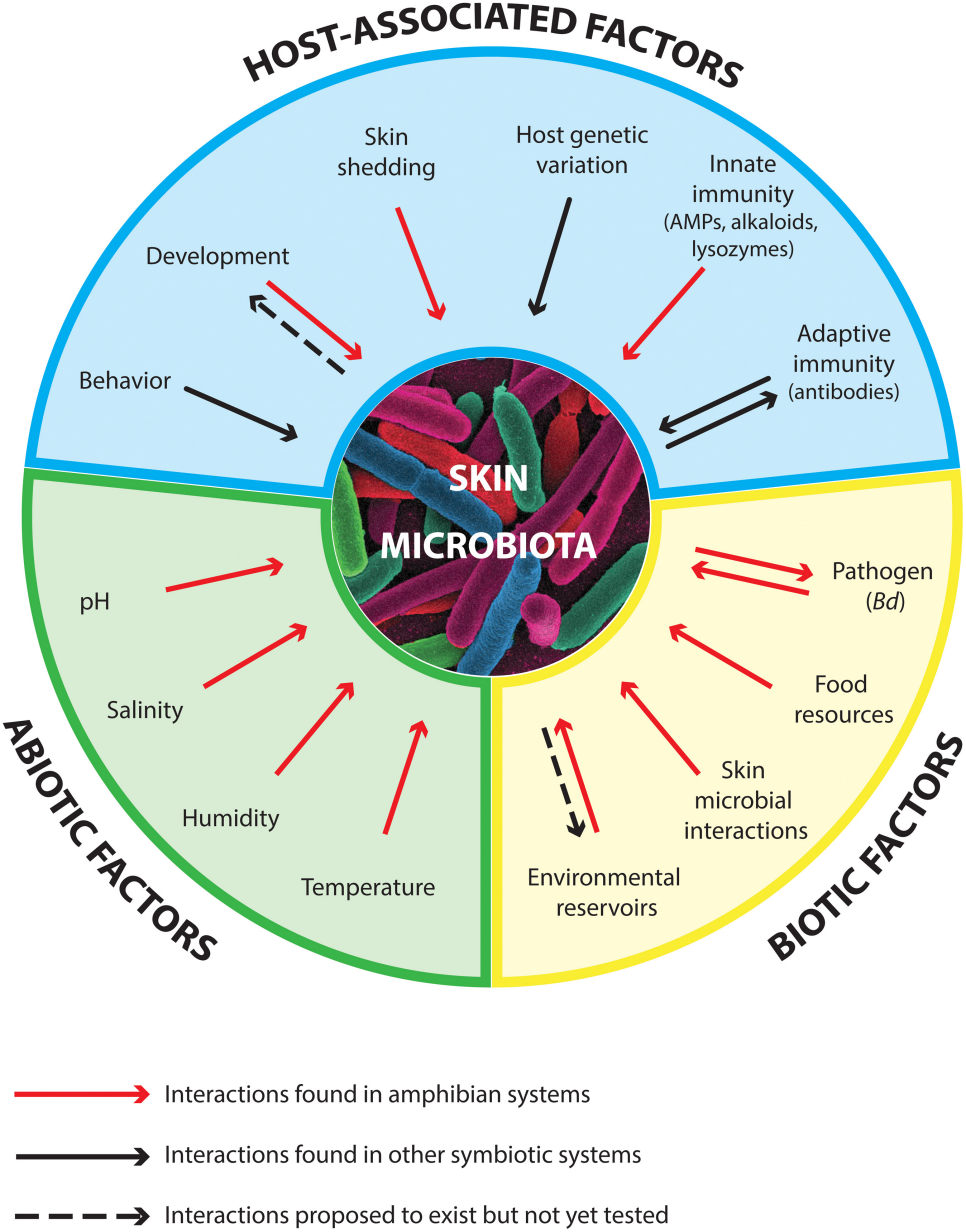
**Main factors that influence the diversity and function of the amphibian skin microbiota, including host-associated factors, biotic factors, and abiotic factors (Box 1)**. Arrows in both directions indicate bidirectional interactions that might occur between the skin microbiota and a particular factor. AMPs stand for antimicrobial peptides. The size of each section is not proportional to the contribution of each of the factors.

The amphibian skin microbiome is determined by the microbiota’s interactions with host-associated factors and with abiotic and biotic factors (**Figure 1**, Box 1). Host-associated factors include host genetic diversity and the adaptive and innate immune systems, in addition to host behavior, ecology and development. Biotic factors include ecological interactions between skin symbiotic microbes and the microbial composition of environmental reservoirs, and abiotic factors include environmental conditions such as temperature and humidity. Altogether, the factors that influence the skin microbiome of amphibians determine the chemical composition of the skin mucus and in turn help determine the degree of host susceptibility against pathogens (Searle et al., 2011; Woodhams et al., 2014). Box 1 summarizes the current state of knowledge on the drivers of the amphibian skin microbiome. We now focus on omics and integrative multi-omics methods and how they can be used to address knowledge gaps that are key to developing effective probiotic strategies.

## Omics Methods to Identify Probiotic Candidates

An important first step toward the identification of potential probiotics in amphibians is to determine differences in the structure and function of skin microbial communities in the presence or absence of *Bd*. This approach includes comparing diseased and not diseased individuals after exposure to *Bd* in laboratory trials, as well as examining *Bd*-tolerant or resistant species from localities that have experienced population declines. The assumption is that individuals or species that persist in the presence of *Bd* might harbor protective bacteria that allowed them to survive. Such studies can be done through 16S amplicon sequencing of the whole microbial community (Caporaso et al., 2012). Furthermore, in order to identify key bacterial species responsible for pathogen protection it will be necessary to go beyond taxonomic descriptions and determine the functional capacities of the community through the use of additional techniques such as shotgun metagenomics, metatranscriptomics, and metabolomics. These approaches can be used to identify bacterial strains that contain genes whose function could make them an effective probiotic. For example, based on previous knowledge about bacterial interactions, searching for genes associated with the production of antibiotics (including anti-fungal metabolites) and beneficial host-microbe interactions could increase the chances of selecting good probiotic candidates.

It is important to emphasize that the use of omic approaches to identify probiotics will only be relevant if these are linked with biological assays of culturable bacteria (*sensu* Becker et al., 2015b). Linking culture-independent with culture-dependent data is a fundamental step toward the identification of successful probiotics (Walke et al., 2015). Importantly, if bacterial cultures with inhibitory activities are available, then physiological, metabolic and genomic analyses of these strains can greatly inform omics predictions. Below, we describe currently available omic approaches and their applications for probiotic selection in amphibians.

### 16S Amplicon Sequencing

Amplicon sequencing is the sequencing of a particular gene or gene fragment of an entire microbial community through the use of high-throughput sequencing methods (Metzker, 2010). In particular, 16S amplicon sequencing of skin bacterial communities has allowed us to determine the most prevalent and relatively abundant bacterial OTUs (operational taxonomic units) on different amphibian species and populations, and across life-history stages (McKenzie et al., 2012; Kueneman et al., 2014; Loudon et al., 2014a; Walke et al., 2014; Rebollar et al., 2016). By providing information about which bacterial taxa appear to be involved in pathogen protection, 16S amplicon sequencing can help target the isolation of potential probiotic bacteria in pure culture. This can be accomplished using data from both field surveys and laboratory experiments.

Field surveys of amphibians naturally exposed to *Bd* can allow the tracking of changes in the microbial community structure in response to *Bd* infection. For example, recent studies in *Rana sierrae* populations have shown a clear correlation between specific OTUs and *Bd* infection intensity in a field survey (Jani and Briggs, 2014). Field studies can therefore inform us about the bacterial taxa that increase in abundance in the presence of *Bd* and might therefore be involved in a concerted response to the infection (Rebollar et al., 2016). This is an essential step to direct the isolation of potential probiotic bacteria in order to test their ability to inhibit *Bd*.

Field surveys, however, are inadequate to determine causal relationships between OTU presence and pathogen presence. Experimental laboratory *Bd* exposures are essential to determine changes in the microbial structure in response to *Bd* infection so they can provide information about which bacterial taxa could be involved in host defense against pathogens. Variation in susceptibility to *Bd* has been linked to changes in cutaneous bacterial community structure (Becker et al., 2015a; Holden et al., 2015), presence of skin antifungal metabolites (Brucker et al., 2008; Becker et al., 2009, 2015b), function of the mucus components (Woodhams et al., 2014), and Major Histocompatibility Complex (MHC) genotype (Savage and Zamudio, 2011; Bataille et al., 2015). For example, an experiment investigating the use of probiotics to prevent chytridiomycosis in the highly susceptible Panamanian golden frog (*Atelopus zeteki*) demonstrated that individuals that were able to clear *Bd* infection harbored a unique community of bacteria on their skin prior to probiotic treatment (Becker et al., 2015a). Furthermore, the authors identified several bacterial families on surviving frogs that were correlated with clearance of *Bd* (Flavobacteriaceae, Sphingobacteriaceae, Comamonadaceae, and Rhodocyclaceae). In contrast OTUs on individuals that died belonged to the families Micrococcineae, Rhizobiaceae, Rhodobacteraceae, Sphingomonadaceae, and Moraxellaceae (Becker et al., 2015a). To determine if the OTUs associated with survival could prevent chytridiomycosis in *A. zeteki*, the next steps would be to isolate the potentially beneficial OTUs from surviving golden frogs, test the isolates for *Bd* inhibition *in vitro* (Bell et al., 2013) and/or use mucosome assays (Woodhams et al., 2014), and finally test the resistance of inoculated individuals to *Bd* infection (Bletz et al., 2013). In addition, the ability to predict host susceptibility via 16S amplicon sequencing may provide a useful tool for captive population managers to identify individuals that could be used for reintroduction trials.

A number of studies using 16S amplicon sequencing have detected bacterial community members that persist independent of variation in environmental reservoirs (Loudon et al., 2014a), time in captivity (Becker et al., 2015a), and in different developmental stages (Kueneman et al., 2014). These prevalent and persistent community members may be closely associated with their hosts over evolutionary timescales. If important for disease defense, OTUs identified in these studies may also provide probiotic candidates that are effective at persisting on hosts, and even naturally transmitted between hosts or across generations (Walke et al., 2011). Augmenting these bacteria in the habitat may also provide disease mitigation benefits (Muletz et al., 2012). Moreover, amplicon sequencing is not limited to bacterial identification but it can also unravel the diversity of micro-eukaryotes through the sequencing of the 18S rRNA gene. For instance, a recent study characterized the bacterial and fungal composition of amphibian skin communities and determined changes in fungal diversity across different developmental stages (Kueneman et al., 2015).

### Shotgun Metagenomics

The sequencing of the total microbial community DNA known as shotgun metagenomics has provided information about the genes present in microbial ecosystems (Barberán et al., 2012b; Knief et al., 2012; Xu et al., 2014). Metagenomic information can allow the identification of genes or genetic pathways associated with specific functions, and therefore it can provide useful information about the potential functional capabilities of microbial communities. For example, metagenomic approaches in marine symbiotic systems have revealed some of the capabilities of bacterial symbionts that are important for interaction with their hosts such as genes involved in nutrient availability and recycling of the host’s waste products (Woyke et al., 2006; Grzymski et al., 2008). As mentioned previously, *in vitro* inhibition assays with bacterial isolates cultured from amphibian skin have detected many bacterial strains with antifungal activities (Harris et al., 2006; Holden et al., 2015; Woodhams et al., 2015). Using metagenomics, these antifungal activities, such as the ability to produce extracellular secondary metabolites, can be identified and bacterial species containing these genes could be inferred. However, metagenomic inferences rely on how much information is available in databases and how much we know about antifungal genetic pathways of isolates in culture. Nonetheless, the comparison of shotgun metagenomic data from resistant and/or tolerant frogs will be very helpful for identifying potential bacterial candidates for probiotics. Bacteria whose genomes contain antifungal gene pathways and pathways associated with the ability to colonize and persist can be identified, which can narrow down the number of probiotic candidates.

### Metatranscriptomics

Metatranscriptomics is the analysis of the mRNA expression profiles in a community and is relevant for identifying genes or genetic pathways that are up or down regulated in response to a pathogen infection. This method can also unravel functional responses involved in bacterial-host interactions such as the expression of adhesin genes or additional traits associated with bacterial colonization and attachment to eukaryotic hosts (Klemm and Schembri, 2000; Dale and Moran, 2006; Kline et al., 2009; Chagnot et al., 2013). Determining the capacity of different bacteria to colonize the skin of the host is extremely relevant for selecting bacterial probiotic candidates. A metatranscriptome approach has shown differences in gene expression in human oral microbiomes between healthy and diseased individuals, and specific metabolic pathways associated with periodontal disease have been identified (Jorth et al., 2014). Metatranscriptomes of fungi and algae in symbiosis with plants and corals, respectively, have also revealed changes in gene expression in response to stressors and environmental cues (Gust et al., 2014; Liao et al., 2014).

Metatranscriptomics may be a good approach in experimental laboratory settings, in which amphibians are exposed to *Bd*. This will allow for the identification of genes that change expression levels in response to pathogen infection and could be associated with host survival. To our knowledge, no studies have used a metatranscriptome approach to study the amphibian skin microbiome, in part because acquiring enough bacterial mRNA from amphibians skin is difficult. To pursue a metatranscriptome approach in amphibians it will be important to improve sampling strategies and molecular methods that increase the bacterial mRNA yield and reduce the proportion of eukaryotic mRNA from the host and from fungi present on the skin (Stewart et al., 2011; Giannoukos et al., 2012; Jorth et al., 2014).

An additional research avenue would be to conduct transcriptomic studies on amphibian hosts (Ellison et al., 2014; Savage et al., 2014; Price et al., 2015) and in parallel measure changes in the skin microbial structure (using 16S amplicon sequencing) and function (metabolomics) in the context of disease or probiotic application. Understanding the role of the genes expressed in host immune responses in shaping the microbiota that colonize and persist on the host may allow novel insights for disease treatment (Box 1). Moreover recent methods like dual RNA-seq, which aim to determine the expression profiles of both the host and the associated microbiota (including pathogens), may allow us to determine the interactions occurring between skin microbiota, the pathogen and the host (Westermann et al., 2012; Schulze et al., 2015). These interactions may provide useful insights for understanding infection dynamics and informing probiotic design.

### Metabolomics

Metabolites are the chemical intermediates and final products of cellular processes, and system-wide attempts to document all chemical species present in a selected biological sample (i.e., metabolomics) have been undertaken for over a decade (Fiehn, 2002; Bouslimani et al., 2015). In amphibians, differences in skin metabolite profiles (representing the sum of host and microbially produced metabolites) across species have been identified (Umile et al., 2014). Metabolomics could also be used to compare species, populations or individuals with varying susceptibility to pathogens like *Bd*. For example, metabolite profiles can be compared between naïve populations and populations that have survived an epidemic of *Bd*, and metabolites that appear among survivors can be identified. The bacteria that produce these metabolites can then be tested for their inhibitory properties and probiotic potential.

A complementary approach is to experimentally expose amphibians to *Bd* and compare the metabolite profiles of survivors and non-survivors. Individuals of the salamander *Plethodon cinereus* that were exposed to *Bd* and survived had significantly higher concentrations of the metabolite violacein on their skins than did individuals that died (Becker et al., 2009). This metabolite is produced by several species of bacteria, most notably *Janthinobacterium lividum*, which lives on the skin of many amphibian species and inhibits *Bd in vitro* (Harris et al., 2006). Moreover, use of *J. lividum* as a probiotic on *Rana muscosa* decreased morbidity when individuals were exposed to *Bd* (Harris et al., 2009), although extension to another host species, the Panamanian golden frog (*A. zeteki*), failed to provide similar protection (Becker et al., 2011).

One challenge in metabolomics is that metabolites vary enormously in chemical structure and reactivity, making the use of a single analytical tool to create a “chemical master inventory” nearly impossible. High-resolution mass spectrometry, often coupled with a separation technique such as high-performance liquid chromatography (LCMS), has led to significant strides in this arena (Dettmer et al., 2007). Since LCMS does not automatically provide molecular structure, further analysis is required, which can involve comparison to molecular databases. Free-access compendia of metabolite data have been published as early as 2005, in the first metabolomics web database METLIN, as well as the subsequent Human Metabolome Database (HMDB; Wishart et al., 2007, 2009, 2013). Another challenge arises from the sheer number of data points generated by such analyses, the visualization of which can be daunting, although multivariate statistical analyses and analytical methods have been presented to address this chemometric challenge (Sharaf et al., 1986; Patti et al., 2013; Bouslimani et al., 2015).

## Statistical Tools to Identify Probiotic Candidates and Integrate Multi-Omics Data

### Identifying Key Bacterial Species Associated with Amphibian Survival Against *Bd*

There are several statistical tools that can be used to identify OTUs that are driving differences at the community level between two or more groups (e.g., susceptible and non-susceptible individuals). For example, indicator species analysis (Dufrene and Legendre, 1997) provides a method to identify indicator OTUs based on the relative abundance and relative frequency of each OTU in predefined groups. In this analysis, each OTU is given an indicator value ranging from one to zero. An OTU that is observed in all the frogs of one group and absent from the other would be designated an indicator value of one. In contrast, an OTU that is equally distributed across both groups would have an indicator value of 0. Statistical significance of each value is then calculated with Monte Carlo simulations. Indicator species analysis can be performed with the IndVal function in the *laBdsv* package (Roberts, 2007) of the R statistical software (R Core Team, 2014).

An additional statistical technique is the Kolmogorov–Smirnov (K–S) Measure (Loftus et al., 2015), which is an extension of the K–S test statistic (Kolmogorov, 1933; Smirnov, 1936). While the K–S test statistic has long been used to assess differences in empirical distribution functions between two groups, the K–S Measure was designed to assess differences in the distributions of the relative abundances of individual OTUs among K > 2 groups. For a given OTU, empirical relative abundance distribution functions are assembled for each group using the data for all individuals assigned to that group. The K–S Measure simultaneously assesses the magnitude of the differences between the distributions, using the weighted sum of the K–S statistics for all pairwise comparisons of distributions defined by K groups. The K–S Measure ranges from zero to one, where values closer to one imply greater differences between the K distributions than values closer to zero (Loftus et al., 2015).

The linear discriminant analysis (LDA) effect size method, LEfSe, can also be an informative method (Segata et al., 2011). LEfSe can be used to compare among groups that are biologically relevant and determine which features (organisms, clades, OTUs, genes, or functions) are significantly different (Albanese et al., 2015; Clemente et al., 2015; Zeng et al., 2015). LEfSe determines the factors that most likely explain differences between classes by coupling standard tests for statistical significance (Kruskal–Wallis and Wilcoxon non-parametric tests) with additional discriminant tests that estimate the magnitude of the effect (LDA score).

Another promising analysis technique is DESeq2, which offers higher power detection for smaller sample sizes (less than 20 samples per group) compared to traditional non-parametric tests based on Kruskal–Wallis and Wilcoxon rank-sum approaches (McMurdie and Holmes, 2014; Weiss et al., 2015). While the non-parametric tests do not assume a distribution, DESeq2 assumes a negative binomial distribution to obtain maximum likelihood estimates for a feature’s (gene, OTU, etc.) log-fold change between two groups (Anders and Huber, 2010; Love et al., 2014). Bayesian shrinkage is then used to reduce the log-fold change toward zero for those OTUs of lower mean count and/or with higher dispersion in their count distribution. These shrunken log-fold changes are tested for significance with a Wald test. If the average number of sequences per sample between the two sample groups differs greatly (>3x), it is better to use a Kruskal–Wallis type approach such as LEfSe for lower type 1 error. All methods, indicator species, K–S Measure, LEfSe and DESeq2, take into account the relative abundance and prevalence of each OTU with the latter two methods allowing for a stratified statistical design with biologically relevant classes and subclasses.

We suggest that all or some of these statistical methods can be used in parallel to identify taxa involved in protection against pathogens. Importantly, some of these statistical tools can be used to identify OTUs based on 16S amplicon sequencing but they can also be used to identify genes and metabolites associated with pathogen protection based on metagenomics, metatranscriptomics, and metabolomics data (Segata et al., 2011; Love et al., 2014; Loftus et al., 2015; Weiss et al., 2015).

### Defining Interactions and Networks Involved in Protection Against Pathogens

One inherent challenge of omic data is interpreting the complex interactions present within the data collected. Many microbial datasets can have more than 5,000 features (e.g., OTUs in the case of 16S amplicon sequencing), so this implies almost 12.5 million possible two-feature correlations. Also, it is expected that within these complex microbial communities three or more feature interactions will occur. Furthermore, omic datasets exhibit diverse challenges, including only providing relative abundances based on a fixed total number of sequences rather than absolute abundances, or the abundance and spatial distribution of zeroes in a data matrix (compositionality; Aitchison, 1986; Lovell et al., 2010; Friedman and Alm, 2012). Data sets with many zeroes, due to incomplete sampling or to ecological interactions (e.g., parasitism, commensalism, etc.), further complicates statistical analysis (Reshef et al., 2011; Friedman and Alm, 2012). However, despite the challenges, computation is possible in terms of time and expense as compared with evaluating more than 12.5 million microbial interactions in the laboratory. Also, the mathematical and statistical approaches for analyzing community data are improving. One technique for inferring microbial interactions from sequencing data is correlation network analysis. Networks consist of “nodes” (OTUs, genes, metabolites, integrated omics) and “edges,” based on the strength of the interaction between nodes, and which imply a biologically or biochemically meaningful relationship between features (Imangaliyev et al., 2015). Interaction values between nodes are commonly referred to as co-occurrence patterns (Faust and Raes, 2012).

Many different techniques have been developed for assessing correlations and constructing interaction networks. Some classic correlation techniques are the Pearson correlation coefficient (Pearson, 1909), which assess linear relationships, or the Spearman correlation coefficient (Spearman, 1904), which measures ranked relationships. Both Pearson and Spearman correlation are very useful (e.g., Arumugam et al., 2011; Barberán et al., 2012a; Buffie et al., 2015), however, neither was developed specifically for the challenges of sequencing data, e.g., compositionality. Of the two, Spearman is less adversely affected by these challenges. Other correlation methods that have been developed include CoNet (Faust et al., 2012), MENA, or Molecular Ecological Network Analysis (Zhou et al., 2011; Deng et al., 2012), Maximal Information Coefficient (MIC; Reshef et al., 2011), Local Similarity Analysis (LSA; Ruan et al., 2006; Beman et al., 2011; Steele et al., 2011; Xia et al., 2013), and Sparse Correlations for Compositional Data (SparCC; Friedman and Alm, 2012). Network visualizations are often performed in the igraph package in R (R Core Team, 2014) or in Cytoscape (Shannon et al., 2003).

**FIGURE 2:**
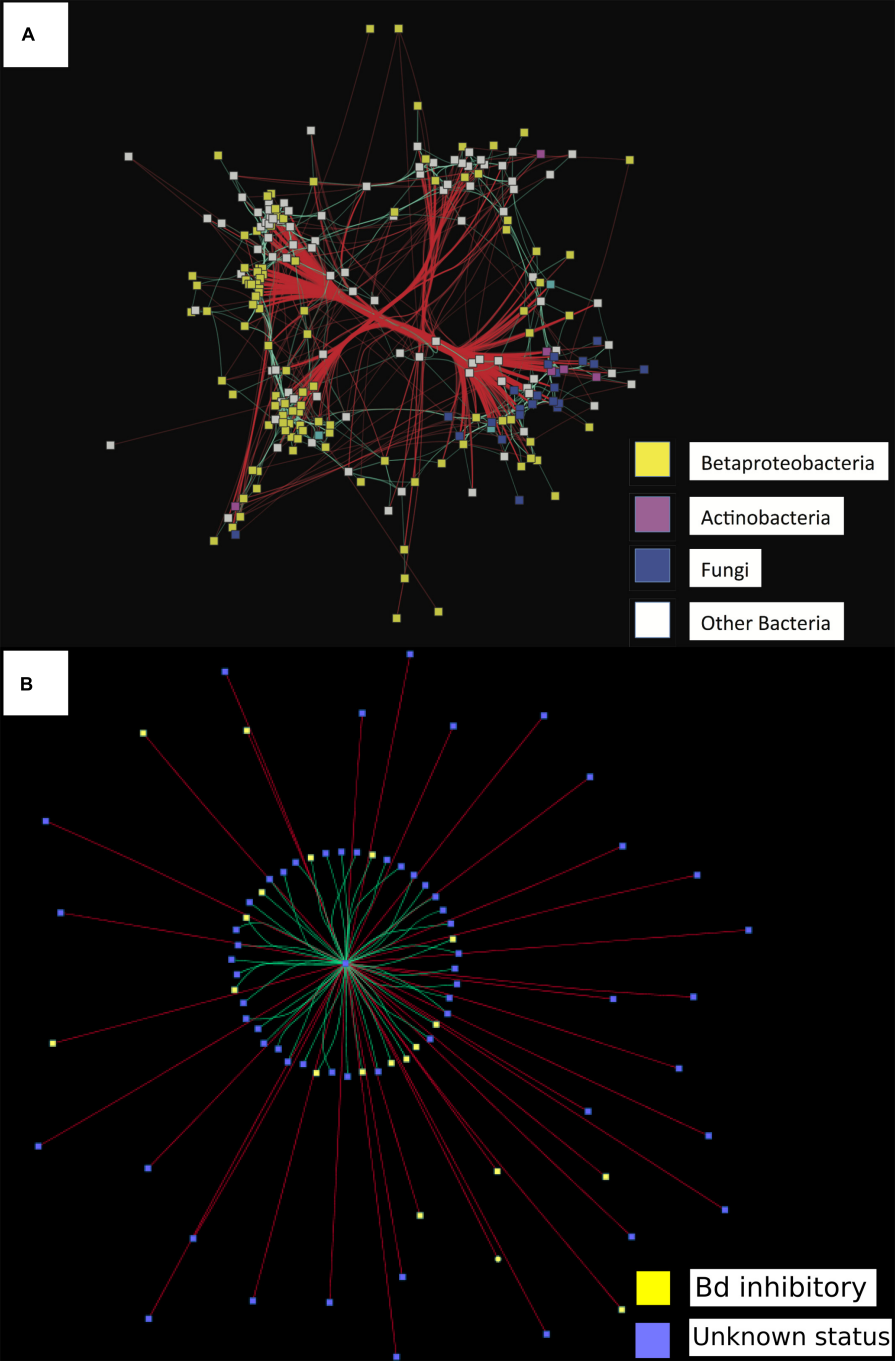
**Data showing proof of concept of a network analysis to identify correlations among bacterial and fungal operational taxonomic units (OTUs) on amphibian hosts**. Network analyses depicting significantly correlated bacterial and fungal OTUs (SparCC *r* > 0.35). All square nodes represent OTUs (either bacteria or fungi). Red lines indicate negative correlations between two OTUs. Turquoise lines indicate positive correlations between two OTUs. **(A)** Assessing directionality of interactions found between all bacteria and fungal taxa. Figure adapted from Kueneman et al. (2015). Yellow = Betaproteobacteria, purple = Actinobacteria, blue = Fungi, white = other bacterial OTUs. **(B)** Assessing directionality of interactions found between all bacterial interactions and Pathogen *Bd*. Yellow = bacteria that inhibit *Bd* in co-culture, blue = Unknown interaction with *Bd* in co-culture. Center of network = fungal pathogen *Bd*.

For probiotic selection, the construction and analysis of networks can infer which taxa occur together in natural communities, and can attempt to identify the direction of interactions between taxa or groups of highly connected taxa (Barberán et al., 2012a). For example, correlation networks in human and mouse models helped identify *Clostridium scindens* as exhibiting a negative correlation pattern with the pathogen *C. difficile*. Transfer of *C. scindens*, either alone or with other bacteria identified by the correlation networks, was then experimentally shown to increase resistance to *C. difficile* infection in mouse models (Buffie et al., 2015). In the case of amphibians, networks that integrate bacterial and fungal omics data taken from hosts, can inform our understanding of interactions occurring between diverse bacterial and fungal taxa (**Figure 2A**). Determining the negative or positive correlations that shift in the presence of a pathogen like *Bd* in experimental trials could help distinguish groups of microbes (mainly bacteria and fungi) involved in resistance against pathogens (**Figure 2B**).

In order to identify potential probiotics against *Bd* in amphibians, correlation networks can be used to compare individual or group interactions in omic data between (1) *Bd*-positive and *Bd*-negative populations in the field, (2) *Bd*-infected and uninfected hosts in experimental trials, (3) hosts with differential *Bd* infection intensity in the field or in experiments and (4) hosts from different life stages. However, caution is warranted when inferring a mechanism of interaction based solely on patterns of correlation (Levy and Borenstein, 2013). Targeted culturing of taxa identified by networks may additionally be used to inform probiotic selection and test their ability to inhibit *Bd* singly or jointly (see Using Omics to Predict if Probiotic Candidates Should be Tested Individually or in Combination), as there may be synergistic *Bd* inhibition (Loudon et al., 2014b). These taxa can be the basis for forming specific hypotheses that can be explored in experimental studies such as a probiotic treatment to determine if the addition of these species to amphibian skin can establish, persist, and increase the anti-*Bd* function of the microbial community of susceptible species.

### Integrating Multi-Omics Data to Identify Anti-Fungal Genetic or Metabolic Pathways

Metagenomics, metatranscriptomics, and metabolomics are important tools to determine molecular pathways present in microbial ecosystems. One of the main goals is integrating these multiple massive data sets to distinguish community patterns associated with a specific function such as host disease resistance. Protection against *Bd* in amphibians is likely achieved by a combination of functional pathways present in the skin microbiome in concert with the host’s immune system. Therefore, the integration of multiple high dimensional datasets using predictive computational approaches such as bioinformatic predictive tools, multi-omic correlations and *in silico* models are key to predict functional outcomes within the skin microbiome (Borenstein, 2012; Langille et al., 2013; McHardy et al., 2013; Meng et al., 2014). One approach termed Reverse Ecology offers a promising way to use high-throughput genomic data to infer ecological interactions from complex biological systems (Levy and Borenstein, 2012, 2014). It involves predicting the metabolic capacity of a biological system (including symbiotic systems) based on metagenomic data through the use of graph-theory based algorithms and genome-scale metabolic networks (Borenstein et al., 2008; Borenstein and Feldman, 2009; Freilich et al., 2009; Levy and Borenstein, 2012; Manor et al., 2014). To date, the amphibian skin microbiome has mainly been described using culture-dependent techniques and 16S amplicon sequencing. The use of these additional techniques may greatly improve our understanding of this microbial system and could allow us to identify fundamental metabolic pathways and ecological networks associated with defense against pathogens like *Bd*. For example, multi-omic correlations of 16S amplicon sequencing and metabolomics (McHardy et al., 2013) may allow us to determine bacterial taxa and metabolites associated with *Bd* inhibition in *Bd*-tolerant species and in individuals exposed to *Bd* in experimental trials.

Moreover, the bacterial taxa that produce the metabolites could be determined by statistical methods that associate metabolite presence with bacterial species’ presence. For example, using random forest with machine learning one can rank microbes by relative contribution (importance; Knights et al., 2011a,b,c; Ditzler and Rosen, 2014). Random forest is an accurate machine-learning multi-category classification algorithm for linking abundances of microbial taxa to physiological states such as metabolite production or immune function (Statnikov et al., 2013). Bacterial species that produce one or more anti-*Bd* metabolites that are associated with survival in a *Bd*-positive environment would be excellent probiotic candidates for bioaugmentation in at-risk populations.

## Using Omics and Integrating Multi-Omics Data to Inform Probiotic Selection Through a Filtering Protocol

**FIGURE 3:**
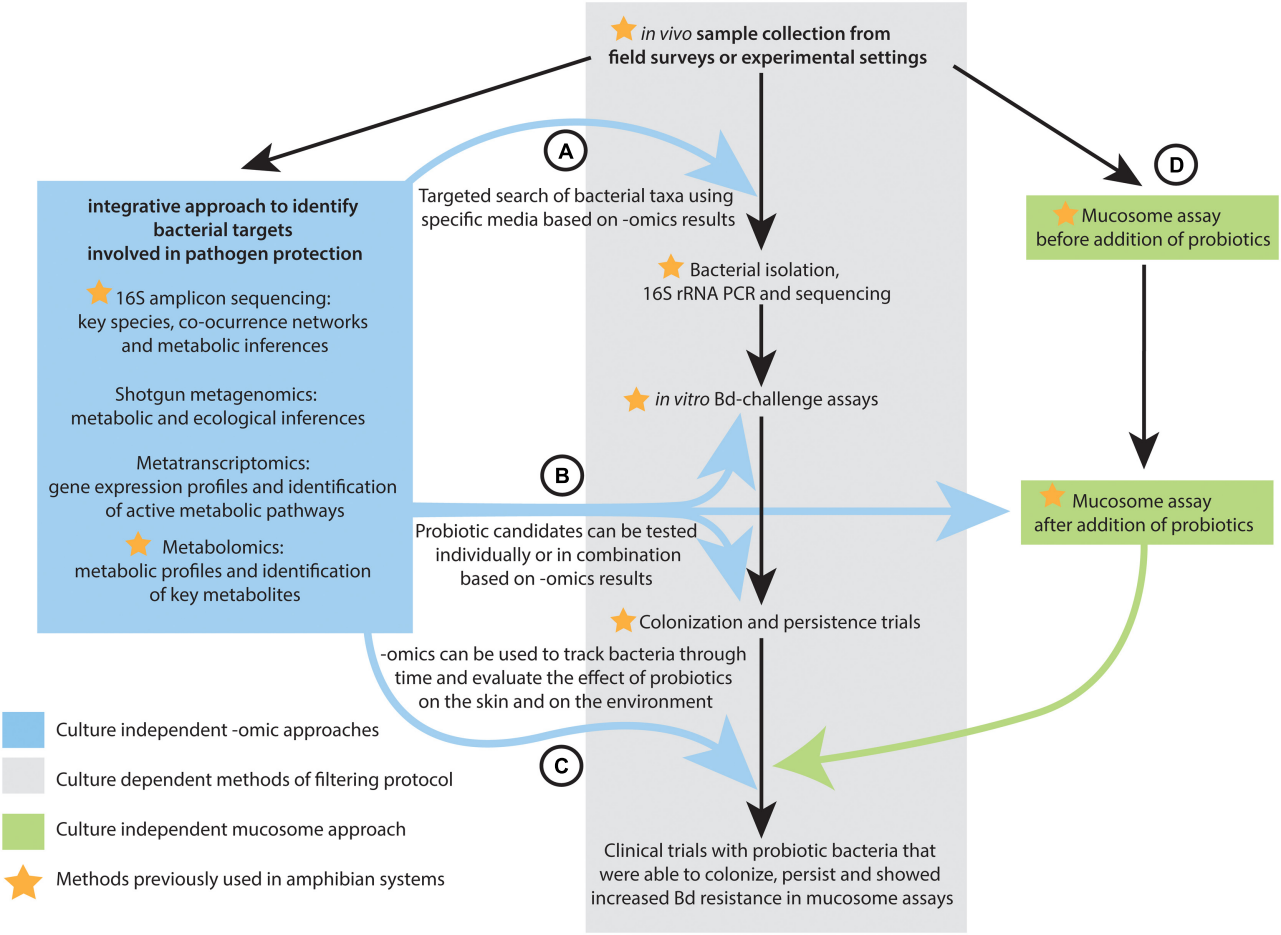
**Flow diagram indicating the steps of the probiotic filtering protocol proposed by Bletz et al. (2013) that can be improved by omics data and integrative multi-omics analyses at different stages: (A)** Integrated multi-omics methods can inform the isolation probiotic candidates, **(B)** Probiotic candidates can be tested individually or in combination based on omics results, and **(C)** Omics approaches can track the effect of probiotic bacteria on the host and on the environment. **(D)** Omics can provide probiotic candidates that can be tested in mucosome assays.

Bletz et al. (2013) recently outlined sampling strategies and screening protocols for identifying ideal probiotics for amphibians (**Figure 3**). The framework involves (1) collecting and culturing skin microbes from selected host species; (2) isolating all morphologically distinct colonies into pure culture; (3) testing each isolate for its ability to inhibit *Bd in vitro* (Bell et al., 2013); (4) testing highly inhibitory isolates for their ability to colonize and persist on amphibian skin; (5) and for those isolates that persist testing their ability to protect the host against *Bd* infection in clinical trials in the laboratory, followed by field trials (Bletz et al., 2013). In the case of the skin of some amphibian species, the dominant members of the microbiota are readily cultured (Walke et al., 2015), whereas some rare but prevalent members identified by 16S amplicon sequencing have been difficult to isolate in culture (Loudon et al., 2014a). Specialized media may be necessary to target microbes identified by omics approaches including not only bacteria but also fungi. Even though most of the probiotic search in amphibians has focused on bacterial candidates, the filtering protocol proposed by Bletz et al. (2013) could be also used to target potential fungal probiotics. Omic datasets and the integration of multi-omic analyses can facilitate the selection of the probiotic candidates that progress through this sampling and screening protocol. Below we describe the steps of the filtering protocol (Bletz et al., 2013) and the mucosome assay (Woodhams et al., 2014) that can be improved by omics and the integration of multi-omic approaches (**Figure 3**).

### Using Omics Data to Inform the Isolation of Probiotic Candidates

A probiotic approach typically requires culturing and isolation of microbial species in order to test their antifungal functions and use only those species with desired properties. Given the taxonomic diversity of the amphibian skin microbiome (Kueneman et al., 2014, 2015; Loudon et al., 2014a; Walke et al., 2014; Becker et al., 2015a), it would be useful to reduce the number of microorganisms that one is trying to isolate and test for inhibition. Omics methods can streamline the isolation process by identifying promising probiotic taxa, which can then be isolated using media and culture conditions that favor or enrich for specific bacterial or fungal groups (Watve et al., 2000; Connon and Giovannoni, 2002; Rappé et al., 2002; Zengler et al., 2002; Vartoukian et al., 2010).

Through the integration of multi-omics data, microbial community members that are associated with surviving amphibian populations in the field and in laboratory experiments can be identified. We suggest using several methods such as indicator species analysis, the K–S Measure, LEfSe and co-occurrence networks in parallel to identify probiotic candidates. The main goal of using several methods is to obtain a list of OTUs that are congruent among methods. OTUs suggested as probiotic candidates by these culture-independent methods can be matched to bacterial isolates in pure culture identified with 16S rRNA Sanger sequencing (Woodhams et al., 2015) or to bacterial strains whose whole genome has been sequenced. These isolates would then proceed to testing for inhibition against *Bd* using *in vitro* challenge assays (Bell et al., 2013) or mucosome assays (Woodhams et al., 2014).

### Using Omics to Predict if Probiotic Candidates should be Tested Individually or in Combination

Data obtained by omics methods can be useful for determining if single species or combinations of species are optimal for probiotic inoculation. Isolates can be chosen based on co-occurrence networks and genetic or metabolic pathways enriched in hosts that survived in the presence of *Bd* or that cleared *Bd* infections.

Single isolate probiotics have been successful in some systems such as the probiotic bacterium *J. lividum* in experimental trials with the host *R. muscosa* experimental trials (Harris et al., 2009). However, research in several symbiotic systems has shown that bacterial mixtures are necessary to exert a protective effect against pathogens and a restorative effect on hosts (Lawley et al., 2012; Fraune et al., 2014). For example, in a mouse model of *C. difficile* infection, a six-species probiotic mixture led to a community reset *and* recovery from *C. difficile* infection (Lawley et al., 2012). The authors speculated that the six species in combination were successful due to their phylogenetic distinctiveness, which allowed them to more effectively fill available niche space. Importantly each species alone was not curative, but each species was necessary in the mixture for the treatment to be effective (Lawley et al., 2012).

We currently do not know when a one-species probiotic or when a mixture will be more effective against *Bd* in amphibians. Omics data might offer insight into why single probiotics have failed in some cases. In addition, the integration of multi-omic data could be used to choose sets of isolates that might work in concert based on the presence of facilitative interactions among them from co-occurrence networks or based on the existence of complementary components of genetic or metabolic pathways.

### Using Omics Data to Track the Effectiveness of Probiotic Bacteria in Laboratory and Field Trials

Omics can be used to determine if a probiotic will be able to colonize, persist and/or trigger antifungal pathways in the symbiotic community. This is key to its success as a probiotic (Bletz et al., 2013). We hypothesize that this can be accomplished if the community reaches an alternative stable state once the candidate taxon is applied and community structure begins to shift in response (Faust and Raes, 2012; Fierer et al., 2012). The new stable state of the community must have antifungal functions and sufficient competitive abilities against invading pathogens to protect the host. Co-occurrence networks could be helpful to track whether bacterial interactions remain stable or shift through time after probiotic application (Rosvall and Bergstrom, 2010). In addition, these approaches could be useful for understanding whether probiotics applied during one life stage persist and remain effective in subsequent life stages (e.g., through metamorphosis).

One of the ultimate goals of probiotic bioaugmentation is for it to be used to reintroduce *Bd*-susceptible amphibian species back into their natural habitats. Thus, candidate probiotics must persist on the host and function appropriately not only in laboratory settings, but also in the host organism’s natural environment. In addition, an ideal probiotic would not disturb other microbial systems, including those of non-target host organisms, upon introduction. This is particularly important to consider if the planned mode of delivery or maintenance of the probiotic is via the soil or water (Muletz et al., 2012). Similar to laboratory trials, it will be important to collect and evaluate “before and after” metagenomic, metatranscriptomic, and metabolomic data to better understand the responses of both the host organism and microbial systems in the surrounding environment to probiotic application.

### Using Omics Approaches to Inform Probiotic Testing in Mucosome Assays

The integrated defenses of the amphibian skin mucus, including antimicrobial peptides, mucosal antibodies, lysozymes and alkaloid secretions and the skin microbiota, are called the mucosome. A mucosome assay developed by Woodhams et al. (2014) can be used to predict the infection prevalence of *Bd*-exposed populations and the survival outcome upon exposure. Briefly, a mucosome assay consists of placing individuals in a bath that collects their mucosal secretions. The secretions are used for *in vitro* viability assays, in which they are tested for their ability to kill *Bd*. This assay accurately predicts adult amphibian survival upon *Bd* exposure (Woodhams et al., 2014).

Importantly, the mucosome assay can be used to measure and predict the effectiveness of probiotic treatments. Probiotic candidates identified by omics analyses need to be isolated through culturing methods and then added to amphibian skins to evaluate their effectiveness using a mucosome assay. This is accomplished by comparing the mucosome function before and after the addition of a probiotic bacterium or a group of probiotic bacteria. Probiotic candidates that pass the preliminary assay screen would then be ready for persistence and clinical trials (**Figure 3D**). The advantage of using the mucosome assay is that it could minimize the need to expose amphibians to *Bd* in clinical trials, which is particularly relevant in the case of endangered species or species that are naïve to the disease.

## Important Considerations and Future Directions

To facilitate the identification of successful probiotic candidates, we recommend using an interdisciplinary approach. The interaction and collaboration of scientists who have different expertise as well as interaction with natural resource managers can greatly improve the outcomes of probiotic research.

In addition to probiotic therapy for the reintroduction of species currently being held in captivity, one important challenge is to identify probiotic candidates for species that are still naïve to pathogen infections. Two relevant cases from highly diverse regions are frogs in regions of Madagascar that have not been exposed to *Bd* (Bletz et al., 2015) and North American salamanders that are so far naïve to *Bsal* (Martel et al., 2013, 2014; Yap et al., 2015).

Omic methods, along with mucosome assays and culture-dependent methods, may greatly improve our knowledge of the capacity of amphibians (and their microbiomes) to contend against pathogenic infections. In addition, other non-omic techniques such as real-time PCR (qPCR), fluorescence *in situ* hybridization (FISH) and mass spectrometry of culturable communities may inform probiotic discovery since they can increase our understanding of the absolute abundances and spatial dynamics of the dominant microbial members in the amphibian skin microbiome (Watrous et al., 2012; Barea, 2015).

Based on previous research on the amphibian system, this review has mainly focused on bacterial probiotics. However, future research may benefit by considering the micro-eukaryotic and viral components of the skin community. Indeed, fungi are important components of mammalian and amphibian skin (Underhill and Iliev, 2014; Kueneman et al., 2015), and viruses have been linked to dysbiosis in the oral cavity (Edlund et al., 2015). Several studies have examined the importance of non-bacterial microbiota in host health (Parfrey et al., 2014; Rizzetto et al., 2015). Indeed, the co-occurrence of diverse microbiota can drive conflicting immune responses and cause trade-offs (Susi et al., 2015).

In addition to altering the microbiota with probiotic bioaugmentation, prebiotics, which are non-digestible carbohydrates, may also have beneficial effects (Patel and Denning, 2013). Prebiotics can alter nutrient sources to selectively favor targeted microbes as in the case of the prebiotics applied in aquaculture systems (Ringø et al., 2010; Akhter et al., 2015). This line of research has only being applied in the intestinal system (Gourbeyre et al., 2011; Patel and Denning, 2013). Thus further research is needed to determine how prebiotics and the combination of probiotics and prebiotics (synbiotics) could be applied to the amphibian system to favor the colonization and growth of antifungal microbes. Moreover, the use of bacterial metabolic products from probiotic microorganisms (postbiotics, Patel and Denning, 2013) and the addition of non-replicating probiotics may lead to effective therapeutics against pathogens.

## Conclusion

Omic methods provide us with the opportunity to thoroughly describe microbial symbiont communities and to determine their structure and functionality. In particular, the skin microbiome in amphibians can be elucidated through the integration of multi-omics data to identify potential key beneficial microbiota and the antifungal genetic and metabolic pathways involved in protection against *Bd* or *Bsal*. Disease mitigation through bioaugmentation can be and has been applied to other biological systems, such as bats fighting against white nose syndrome disease, as well as in cattle raising, agricultural and aquacultural systems (Kesarcodi-Watson et al., 2008; Bhardwaj et al., 2014; Lakshmanan et al., 2014; Hoyt et al., 2015; Papadimitriou et al., 2015; Uyeno et al., 2015). These systems share similar concerns and also have the difficulties that we have described here in finding mitigation solutions, so they could benefit from this omics approach. We have a clear framework for selecting an ideal probiotic (Bletz et al., 2013); however, integrative multi-omics can prioritize candidates and facilitate selection of candidates to move to the next steps in the filtering protocol. Finding effective probiotics has the potential to reduce the large losses of biodiversity from EIDs such as chytridiomycosis.

### Box 1. Factors Influencing the Amphibian Skin Microbiome

#### Host-Associated Factors: Genetic and Immune System Diversity

Due to the essential function of amphibian skin in protection of the host against desiccation and pathogens, the skin mucus is a niche with unique chemical properties. Thus, one would predict that only a limited subset of bacterial species would be able to become established on the host (habitat filtering). However, the extent to which amphibian host factors dictate the selection, diversity, and stability of the skin microbiota remains poorly understood. Moreover, we still lack knowledge about how much variation in microbial community structure can be supported by host amphibian genotypes. In other animals, it is clear that many host-specific factors can regulate the assembly of their microbial communities. For example, previous studies in humans and laboratory mice have shown that different genotypes support different microbiota (reviewed in Spor et al., 2011). Likewise, in *Nasonia* wasps, ants and freshwater *Hydra*, species-specific microbiota emerge in “phylosymbiotic” patterns that parallel speciation and ancestry (Brucker and Bordenstein, 2012, 2013; Franzenburg et al., 2013; Sanders et al., 2014). In other animal systems genetic variation of immune associated genes has been associated with differences in symbiotic microbiota. For example, the gut microbial community structure of sticklebacks (*Gasterosteus aculeatus*) is correlated with the diversity of the individual’s MHC Class IIb genes (Bolnick et al., 2014). The MHC is a major set of adaptive immune genes that code for molecules that regulate recognition of foreign antigens and pathogens; however, the role of the MHC in regulating microbial communities is poorly understood. A proposed mechanism for MHC control of microbiota is that some MHC molecules may vary in their capacity to recognize the microbial motifs needed to mount an immune response against particular bacterial taxa (Bolnick et al., 2014). Microbes or their microbial antigens are taken up by antigen presenting cells such as dendritic cells and macrophages. The processed antigens are then presented as small peptides complexed with MHC to T lymphocytes. The T lymphocytes release cytokines that recruit other effector cells, and they assist the development of antibodies.

In amphibians, several studies have demonstrated that the host microbiota in amphibians can vary by population (McKenzie et al., 2012; Kueneman et al., 2014; Walke et al., 2014), and it is possible that these differences correlate with population-level differences in immunogenetic diversity. Because the mucus of amphibians contains several classes of antibodies (Ramsey et al., 2010), it is likely that the antibodies expressed in the mucus would play a role in controlling which microbial species are allowed to colonize (Colombo et al., 2015).

In addition to the genetic diversity of genes involved in the adaptive immune system, amphibians produce a diverse array of innate immune defenses including antimicrobial peptides (AMPs), lysozymes and alkaloids (Macfoy et al., 2005; Conlon, 2011). A diverse array of AMPs are produced in amphibian’s granular glands such as brevinins, ranatuerins, and magainins that are encoded by polymorphic genes that generate variation in peptide profiles among individuals (Tennessen and Blouin, 2007; Tennessen et al., 2009; Conlon, 2011; Daum et al., 2012). In comparison with the genetic diversity of MHC molecules, AMP genes and expressed peptides are much less diverse (Tennessen and Blouin, 2007). However, apparent gene duplications allow for gradual genetic changes that appear to be positively selected in response to pathogens (Tennessen and Blouin, 2007). Defensive AMPs appear to be released constitutively into the mucus at low concentrations, but they can be increased significantly when the amphibian hosts are alarmed or injured (Pask et al., 2012) and can be affected by environmental stressors (Katzenback et al., 2014). However, some amphibians appear to lack the capacity to produce conventional cationic AMPs (Conlon, 2011). Species that lack AMPs may be more dependent on other chemical factors present in the mucus (bacterial antifungal metabolites, lysozymes, and antibodies) in order to mount a defense against skin pathogens.

In summary, all of the host mucosal chemical defenses (AMPs, lysozyme, alkaloids, and antibodies) have the potential to affect survival of some members of the community of skin bacteria. The interplay between chemical defenses in the mucus and microbial communities is not well understood. Future research is needed to understand to what extent microbes shape the immune compartment and how the immune compartment shapes the microbiome.

#### Biotic Factors

Hosts are in constant contact with environmental microbial communities that serve as reservoirs. In the case of amphibian skin microbiota, environmental reservoirs may provide an important source of bacterial colonizers, which are needed since amphibian cutaneous microbial communities are frequently disturbed by skin shedding (Meyer et al., 2012). In humans, bacteria are found in deep epidermal layers, not only on the skin external layer (Nakatsuji et al., 2013), thus providing a reservoir for re-inoculating the skin after disturbance. This has not yet been demonstrated for amphibians; however, the salamander gut has been shown to be a reservoir for the anti-fungal cutaneous bacterium *J. lividum* (Wiggins et al., 2011) and bacteria residing in gland openings may also serve as a reservoir (Lauer et al., 2007).

Nonetheless, environmental reservoirs appear to be necessary to maintain the diversity of skin symbiotic bacteria (Loudon et al., 2014a). For example, salamanders (*P. cinereus*) without an environmental bacterial reservoir showed a 75% decrease in bacterial richness, and their bacterial communities became uneven with some OTUs becoming dominant in the majority of the individuals. In contrast, salamanders that were housed with a soil reservoir maintained a greater bacterial diversity that was more similar to communities found in nature (Loudon et al., 2014a). In the case of red-eyed tree frogs (*Agalychnis callidryas*), individuals housed with plants had a greater richness and abundance of skin bacteria than those housed without plants (Michaels et al., 2014). These studies demonstrate that environmental reservoirs are necessary to maintain the bacterial diversity typically found in nature. In terms of probiotics, Muletz et al. (2012) demonstrated that *P. cinereus* can acquire the beneficial bacterium *J. lividum* from soil, and salamanders that were able to acquire *J. lividum* from the environment were less likely to be infected with *Bd (*Muletz et al., 2012).

The skin microbiota also interacts with invading skin pathogens such as *Bd*. In amphibians the skin mucus contains a suite of microorganisms that may play a beneficial symbiotic role for the host (Harris et al., 2006). Anti-*Bd* secretions from skin bacteria have been found on free-living hosts in concentrations that inhibit *Bd in vitro* (Brucker et al., 2008; Becker et al., 2009). Furthermore, some bacterially produced metabolites interact synergistically and additively to inhibit *Bd* (Myers et al., 2012; Loudon et al., 2014a). Recent work has demonstrated that the composition and structure of amphibian skin bacterial communities can change in response to *Bd* infection (Jani and Briggs, 2014). However, it is still not well understood if changes in the diversity of the microbiota are accompanied by changes in function (i.e., increases in the number of beneficial anti-*Bd* symbionts and therefore an increased protective role of the skin microbiota).

A number of different *Bd* lineages have been identified and isolated from amphibian skin (Farrer et al., 2011; Schloegel et al., 2012; Bataille et al., 2013), including the globally distributed and hypervirulent global panzootic lineage (*Bd*GPL) that has been associated with mass mortalities and rapid population declines of amphibians (Farrer et al., 2011, 2013). Within the *Bd*GPL lineage there is considerable genetic variation, as well as significant differences in virulence between isolates (Farrer et al., 2011, 2013). Many bacterial strains isolated from amphibian skin have the ability to inhibit the growth of *Bd in vitro* (Harris et al., 2006; Woodhams et al., 2014; Becker et al., 2015b; Holden et al., 2015). However, it was recently demonstrated that bacteria differ in their capacity to inhibit different *Bd*GPL isolates, and only a small proportion of bacteria show broad scale inhibition across the genetic variation exhibited by *Bd*GPL (Antwis et al., 2015). This, coupled with the variation in host response to different isolates of *Bd*GPL (Farrer et al., 2011), means that potential probiotics will need to account for differing virulence of *Bd* or that probiotics that show broad scale inhibition must be identified and used.

In addition to the influence of environmental microbes and pathogens, the ecological interactions among skin microbes would be expected to play a relevant role in structuring the skin microbiota. Bacteria engage in the full breadth of ecological interactions, from antagonistic to facilitative (reviewed by Faust and Raes, 2012). Many of these bacterial interactions are chemically mediated by secondary metabolites, which can contribute to mutualistic interactions, such as cross-feeding or syntrophy, in which two species benefit from each other’s metabolic products (Woyke et al., 2006; Faust and Raes, 2012; Loudon et al., 2014b). Secondary metabolite production is also influenced by the composition of the bacterial community (Onaka et al., 2011), and therefore changes in the community composition of the host (for example through environmental variation or diet) may intrinsically lead to changes in the secondary metabolite profile of the total community. In addition to mutualistic interactions, competition is common among microbes, and can occur via antibiotic production (Kelsic et al., 2015). Other forms of competition can range from occupying space and therefore inhibiting attachment of colonizing species to more efficient consumption of shared resources.

#### Abiotic Factors

The skin microbiome in vertebrates is highly sensitive to changes in humidity and temperature (Grice and Segre, 2011; Kueneman et al., 2014). Therefore, the skin microbial community structure might be modified by exposure of the skin to different microclimates. This is particularly relevant for ectotherms like amphibians, in which habitat-mediated thermoregulation can expose the host (and its microbial symbionts) to a wide variety of microclimatic conditions over very short time periods (Huey, 1991). Moreover, seasonal variation may influence host behavior by increasing host body temperature (Rowley and Alford, 2013) and this could in turn modify the skin microbial structure. Warmer temperatures can increase the skin sloughing frequency of anurans, thus reducing the abundance of bacteria on the skin through frequent disturbance (Meyer et al., 2012; Ohmer et al., 2014). In addition, thermal conditions influence the activity and production of antifungal metabolites by symbiotic microbes on amphibian skin (Woodhams et al., 2014). For example, high temperatures can limit the production of antimicrobial metabolites, such as violacein and prodigiosin produced by *J. lividum* strains (Schloss et al., 2010; Woodhams et al., 2014). However, for other bacterial probiotics, cooler temperatures may limit the production of antimicrobial products (Daskin et al., 2014). The combined influences of environmental variation on microbiome stability are poorly understood, and they likely vary among species from different habitats and ecosystems.

Moreover, temperature might also impact the skin microbiome by altering the interaction between the amphibian immune system and invading pathogens. Immunity in ectotherms is strongly affected by temperature (Raffel et al., 2006; Rollins-Smith et al., 2011; Rollins-Smith and Woodhams, 2012). In general, low temperatures (4–10°C) are predicted to favor *Bd* (Woodhams et al., 2008; Voyles et al., 2012), and under these conditions amphibian immune defenses are delayed or diminished (Rollins-Smith et al., 2011; Rollins-Smith and Woodhams, 2012). In contrast, higher temperatures (25–30°C), nearer to the maximum for *Bd* survival (Piotrowski et al., 2004; Stevenson et al., 2013), are predicted to favor the amphibian host, enabling them to develop a more effective immune response (Rowley and Alford, 2013). Similarly, *Bsal* infections can be cleared by host exposure to 25°C for 10 days (Blooi et al., 2015). Thus, thermal preference of the host is associated with lower probabilities for *Bd* or *Bsal* infection (Rowley and Alford, 2013). In this respect, the skin microbiome may also be affected by pathogen invasions based on the host’s and the pathogen’s thermal preferences.

## Author Contributions

RH and ER contributed the original idea and outline. ER, RA, LB, MHB, MCB, RB, XH, AL, DM, KM, LR-S, JW, DW, and RH contributed the initial writing of specific sections. ER, LB, MHB, MH, JK, VM, LR-S, SW, DW, and RH contributed additional relevant ideas and sections as well as structuring the manuscript. ER integrated all sections and produced all drafts of the manuscript, and all authors edited several versions of the manuscript.

## Conflict of Interest Statement

The authors declare that the research was conducted in the absence of any commercial or financial relationships that could be construed as a potential conflict of interest.

## References

[B1] Aitchison J. (1986). *The Statistical Analysis of Compositional Data, Monographs on Statistics and Applied Probability.* London: Chapman & Hall Ltd.

[B2] Akhter N. Wu B. Memon A. M. Mohsin M. (2015). Probiotics and prebiotics associated with aquaculture: a review. *Fish Shellfish Immunol.* 45 733–741. 10.1016/j.fsi.2015.05.038 26044743

[B3] Albanese D. De Filippo C. Cavalieri D. Donati C. (2015). Explaining diversity in metagenomic datasets by phylogenetic-based feature weighting. *PLoS Comput. Biol.* 11:e1004186 10.1371/journal.pcbi.1004186 PMC4376673 25815895

[B4] Altelaar A. F. M. Munoz J. Heck A. J. R. (2012). Next-generation proteomics: towards an integrative view of proteome dynamics. *Nat. Rev. Genet.* 14 35–48. 10.1038/nrg3356 23207911

[B5] Anders S. Huber W. (2010). Differential expression analysis for sequence count data. *Genome Biol.* 11:R106 10.1186/gb-2010-11-10-r106 PMC3218662 20979621

[B6] Antwis R. E. Preziosi R. F. Harrison X. A. Garner T. W. J. (2015). Amphibian symbiotic bacteria do not show universal ability to inhibit growth of the global pandemic lineage of *Batrachochytrium dendrobatidis*. *Appl. Environ. Microbiol.* 81 3706–3711. 10.1128/AEM.00010-15 25819964 PMC4421062

[B7] Arumugam M. Raes J. Pelletier E. Paslier D. Le Batto J. Yamada T. (2011). Enterotypes of the human gut microbiome. *Nature* 473 174–180. 10.1038/nature09944 21508958 PMC3728647

[B8] Barberán A. Bates S. T. Casamayor E. O. Fierer N. (2012a). Using network analysis to explore co-occurrence patterns in soil microbial communities. *ISME J.* 6 343–351. 10.1038/ismej.2011.119 21900968 PMC3260507

[B9] Barberán A. Fernández-Guerra A. Bohannan B. J. M. Casamayor E. O. (2012b). Exploration of community traits as ecological markers in microbial metagenomes. *Mol. Ecol.* 21 1909–1917. 10.1111/j.1365-294X.2011.05383.x 22121910

[B10] Barea J. M. (2015). Future challenges and perspectives for applying microbial biotechnology in sustainable agriculture based on a better understanding of plant-microbiome interactions. *J. Soil Sci. Plant Nutr.* 15 261–282. 10.4067/S0718-95162015005000021

[B11] Bataille A. Cashins S. D. Grogan L. Skerratt L. F. Hunter D. Mcfadden M. (2015). Susceptibility of amphibians to chytridiomycosis is associated with MHC class II conformation. *Proc. R. Soc. B Biol. Sci.* 282:20143127 10.1098/rspb.2014.3127 PMC4389617 25808889

[B12] Bataille A. Fong J. J. Cha M. Wogan G. O. U. Baek H. J. Lee H. (2013). Genetic evidence for a high diversity and wide distribution of endemic strains of the pathogenic chytrid fungus *Batrachochytrium dendrobatidis* in wild Asian amphibians. *Mol. Ecol.* 22 4196–4209. 10.1111/mec.12385 23802586

[B13] Becker M. H. Brucker R. M. Schwantes C. R. Harris R. N. Minbiole K. P. C. (2009). The bacterially produced metabolite violacein is associated with survival of amphibians infected with a lethal fungus. *Appl. Environ. Microbiol.* 75 6635–6638. 10.1128/AEM.01294-09 19717627 PMC2772424

[B14] Becker M. H. Harris R. N. Minbiole K. P. C. Schwantes C. R. Rollins-Smith L. A. Reinert L. K. (2011). Towards a better understanding of the use of probiotics for preventing chytridiomycosis in Panamanian golden frogs. *Ecohealth* 8 501–506. 10.1007/s10393-012-0743-0 22328095

[B15] Becker M. H. Walke J. B. Cikanek S. Savage A. E. Mattheus N. Santiago C. N. (2015a). Composition of symbiotic bacteria predicts survival in Panamanian golden frogs infected with a lethal fungus. *Proc. R. Soc. B* 282:20142881 10.1098/rspb.2014.2881 PMC4389611 25788591

[B16] Becker M. H. Walke J. B. Murrill L. Woodhams D. C. Reinert L. K. Rollins-Smith L. A. (2015b). Phylogenetic distribution of symbiotic bacteria from Panamanian amphibians that inhibit growth of the lethal fungal pathogen *Batrachochytrium dendrobatidis*. *Mol. Ecol.* 24 1628–1641. 10.1111/mec.13135 25737297

[B17] Bell S. C. Alford R. A. Garland S. Padilla G. Thomas A. D. (2013). Screening bacterial metabolites for inhibitory effects against *Batrachochytrium dendrobatidis* using a spectrophotometric assay. *Dis. Aquat. Organ.* 103 77–85. 10.3354/dao02560 23482387

[B18] Beman J. M. Steele J. A. Fuhrman J. A. (2011). Co-occurrence patterns for abundant marine archaeal and bacterial lineages in the deep chlorophyll maximum of coastal California. *ISME J.* 5 1077–1085. 10.1038/ismej.2010.204 21228895 PMC3146281

[B19] Berger L. Speare R. Daszak P. Green D. E. Cunningham A. A. Goggin C. L. (1998). Chytridiomycosis causes amphibian mortality associated with population declines in the rain forests of Australia and Central America. *Proc. Natl. Acad. Sci. U.S.A.* 95 9031–9036. 10.1073/pnas.95.15.9031 9671799 PMC21197

[B20] Berlec A. (2012). Novel techniques and findings in the study of plant microbiota: search for plant probiotics. *Plant Sci.* 19 96–102. 10.1016/j.plantsci.2012.05.010 22794922

[B21] Bhardwaj D. Ansari M. Sahoo R. Tuteja N. (2014). Biofertilizers function as key player in sustainable agriculture by improving soil fertility, plant tolerance and crop productivity. *Microb. Cell Fact.* 13:66 10.1186/1475-2859-13-66 PMC4022417 24885352

[B22] Blehert D. S. Hicks A. C. Behr M. Meteyer C. U. Berlowskizier B. M. Buckles E. L. (2009). Bat white-nose syndrome: an emerging pathogen? *Science* 323:227 10.1126/science.1163874 18974316

[B23] Bletz M. C. Loudon A. H. Becker M. H. Bell S. C. Woodhams D. C. Minbiole K. P. C. (2013). Mitigating amphibian chytridiomycosis with bioaugmentation: characteristics of effective probiotics and strategies for their selection and use. *Ecol. Lett.* 16 807–820. 10.1111/ele.12099 23452227

[B24] Bletz M. C. Rosa G. M. Crottini A. Courtois E. A. Schmeller D. S. Rabibisoa N. E. A. (2015). Widespread presence of the pathogenic fungus *Batrachochytrium dendrobatidis* in wild amphibian communities in Madagascar. *Nat. Commun.* 5:8633 10.1038/srep08633 PMC4341422 25719857

[B25] Blooi M. Martel A. Haesebrouck F. Vercammen F. Bonte D. Pasmans F. (2015). Treatment of urodelans based on temperature dependent infection dynamics of *Batrachochytrium salamandrivorans*. *Sci. Rep.* 4 35–38. 10.1038/srep08037 PMC5389025 25623498

[B26] Bolnick D. I. Snowberg L. K. Caporaso J. G. Lauber C. Knight R. Stutz W. E. (2014). Major histocompatibility complex class IIb polymorphism influences gut microbiota composition and diversity. *Mol. Ecol.* 23 4831–4845. 10.1111/mec.12846 24975397

[B27] Borenstein E. (2012). Computational systems biology and in silico modeling of the human microbiome. *Brief. Bioinform.* 13 769–780. 10.1093/bib/bbs022 22589385

[B28] Borenstein E. Feldman M. W. (2009). Topological signatures of species interactions in metabolic networks. *J. Comput. Biol.* 16 191–200. 10.1089/cmb.2008.06TT 19178139 PMC3035845

[B29] Borenstein E. Kupiec M. Feldman M. W. Ruppin E. (2008). Large-scale reconstruction and phylogenetic analysis of metabolic environments. *Proc. Natl. Acad. Sci. U.S.A.* 105 14482–14487. 10.1073/pnas.0806162105 18787117 PMC2567166

[B30] Bouslimani A. Porto C. Rath C. M. Wang M. Guo Y. Gonzalez A. (2015). Molecular cartography of the human skin surface in 3D. *Proc. Natl. Acad. Sci. U.S.A.* 12 E2120–E2129. 10.1073/pnas.1424409112 25825778 PMC4418856

[B31] Brucker R. M. Bordenstein S. R. (2012). The roles of host evolutionary relationships (genus: *Nasonia*) and development in structuring microbial communities. *Evolution* 66 349–362. 10.1111/j.1558-5646.2011.01454.x 22276533

[B32] Brucker R. M. Bordenstein S. R. (2013). The hologenomic basis of speciation: gut bacteria cause hybrid lethality in the genus *Nasonia*. *Science* 341 667–669. 10.1126/science.1240659 23868918

[B33] Brucker R. M. Harris R. N. Schwantes C. R. Gallaher T. N. Flaherty D. C. Lam B. A. (2008). Amphibian chemical defense: antifungal metabolites of the microsymbiont *Janthinobacterium lividum* on the salamander *Plethodon cinereus*. *J. Chem. Ecol.* 34 1422–1429. 10.1007/s10886-008-9555-7 18949519

[B34] Buffie C. G. Bucci V. Stein R. R. McKenney P. T. Ling L. Gobourne A. (2015). Precision microbiome reconstitution restores bile acid mediated resistance to *Clostridium difficile*. *Nature* 517 205–208. 10.1038/nature13828 25337874 PMC4354891

[B35] Caporaso J. G. Lauber C. L. Walters W. A. Berg-Lyons D. Huntley J. Fierer N. (2012). Ultra-high-throughput microbial community analysis on the Illumina HiSeq and MiSeq platforms. *ISME J.* 6 1621–1624. 10.1038/ismej.2012.8 22402401 PMC3400413

[B36] Caporaso J. G. Lauber C. L. Walters W. A. Berg-Lyons D. Lozupone C. A. Turnbaugh P. J. (2011). Global patterns of 16S rRNA diversity at a depth of millions of sequences per sample. *Proc. Natl. Acad. Sci. U.S.A.* 108(Suppl.1), 4516–4522. 10.1073/pnas.1000080107 20534432 PMC3063599

[B37] Chagnot C. Zorgani M. A. Astruc T. Desvaux M. (2013). Proteinaceous determinants of surface colonization in bacteria: bacterial adhesion and biofilm formation from a protein secretion perspective. *Front. Microbiol.* 4:303 10.3389/fmicb.2013.00303 PMC3796261 24133488

[B38] Clemente J. C. Pehrsson E. C. Blaser M. J. Sandhu K. Gao Z. Wang B. (2015). The microbiome of uncontacted Amerindians. *Sci. Adv.* 1:e1500183 10.1126/sciadv.1500183 PMC4517851 26229982

[B39] Colombo B. M. Scalvenzi T. Benlamara S. Pollet N. (2015). Microbiota and mucosal immunity in amphibians. *Front. Immunol.* 6:111 10.3389/fimmu.2015.00111 PMC4358222 25821449

[B40] Conlon J. M. (2011). Structural diversity and species distribution of host-defense peptides in frog skin secretions. *Cell. Mol. Life Sci.* 68 2303–2315. 10.1007/s00018-011-0720-8 21560068 PMC11114843

[B41] Connon S. A. Giovannoni S. J. (2002). High-throughput methods for culturing microorganisms in very-low-nutrient media yield diverse new marine isolates. *Appl. Environ. Microbiol.* 68 3878–3885. 10.1128/AEM.68.8.3878 12147485 PMC124033

[B42] Dale C. Moran N. A. (2006). Molecular interactions between bacterial symbionts and their hosts. *Cell* 126 453–465. 10.1016/j.cell.2006.07.014 16901780

[B43] Daskin J. H. Bell S. C. Schwarzkopf L. Alford R. A. (2014). Cool temperatures reduce antifungal activity of symbiotic bacteria of threatened amphibians - implications for disease management and patterns of decline. *PLoS ONE* 9:e100378 10.1371/journal.pone.0100378 PMC4062522 24941262

[B44] Daum J. M. Davis L. R. Bigler L. Woodhams D. C. (2012). Hybrid advantage in skin peptide immune defenses of water frogs (*Pelophylax esculentus*) at risk from emerging pathogens. *Infect. Genet. Evol.* 12 1854–1864. 10.1016/j.meegid.2012.07.024 22940461

[B45] Deng Y. Jiang Y.-H. Yang Y. He Z. Luo F. Zhou J. (2012). Molecular ecological network analyses. *BMC Bioinformatics* 13:113 10.1186/1471-2105-13-113 PMC3428680 22646978

[B46] Dettmer K. Aronov P. A. Hammock B. D. (2007). Mass spectrometry-based metabolomics. *Mass Spectrom. Rev.* 26 51–78. 10.1002/mas.20108 16921475 PMC1904337

[B47] Ditzler G. Rosen G. (2014). Feature subset selection for inferring relative importance of taxonomy,” in *Proceedings of the 5th ACM Conference on Bioinformatics, Computational Biology, and Health Informatics*, New York, NY, 673–679.

[B48] Dufrene M. Legendre P. (1997). Species assemblages and indicator species: the need for a flexible asymmetrical approach. *Ecol.* *Monogr.* 67 345–366. 10.1890/0012-9615(1997)067[0345:SAAIST]2.0.CO;2

[B49] Edlund A. Santiago-Rodriguez T. M. Boehm T. K. Pride D. T. (2015). Bacteriophage and their potential roles in the human oral cavity. *J. Oral Microbiol.* 7 1–12. 10.3402/jom.v7.27423 PMC4393417 25861745

[B50] Ellison A. R. Tunstall T. DiRenzo G. V. Hughey M. C. Rebollar E. A. Belden L. K. (2014). More than skin deep: functional genomic basis for resistance to amphibian chytridiomycosis. *Genome Biol. Evol.* 7 286–298. 10.1093/gbe/evu285 25539724 PMC4316636

[B51] Farrer R. A. Henk D. A. Garner T. W. J. Balloux F. Woodhams D. C. Fisher M. C. (2013). Chromosomal copy number variation, selection and uneven rates of recombination reveal cryptic genome diversity linked to pathogenicity. *PLoS Genet.* 9:e1003703 10.1371/journal.pgen.1003703 PMC3744429 23966879

[B52] Farrer R. A. Weinert L. Bielby J. Garner T. W. J. Balloux F. Clare F. (2011). Multiple emergences of genetically diverse amphibian-infecting chytrids include a globalized hypervirulent recombinant lineage. *Proc. Natl. Acad. Sci. U.S.A.* 108 18732–18736. 10.1073/pnas.1111915108 22065772 PMC3219125

[B53] Faust K. Raes J. (2012). Microbial interactions: from networks to models. *Nat. Rev. Microbiol.* 10 538–550. 10.1038/nrmicro2832 22796884

[B54] Faust K. Sathirapongsasuti J. F. Izard J. Segata N. Gevers D. Raes J. (2012). Microbial co-occurrence relationships in the Human Microbiome. *PLoS Comput. Biol.* 8:e1002606 10.1371/journal.pcbi.1002606 PMC3395616 22807668

[B55] Fiehn O. (2002). Metabolomics - the link between genotypes and phenotypes. *Plant Mol. Biol.* 48 155–171. 10.1023/A:1013713905833 11860207

[B56] Fierer N. Ferrenberg S. Flores G. E. González A. Kueneman J. Legg T. (2012). From animalcules to an ecosystem: application of ecological concepts to the Human Microbiome. *Annu. Rev. Ecol. Evol. Syst.* 43 137–155. 10.1146/annurev-ecolsys-110411-160307

[B57] Fisher M. C. Garner T. W. J. Walker S. F. (2009). Global emergence of *Batrachochytrium dendrobatidis* and amphibian chytridiomycosis in space, time, and host. *Annu. Rev. Microbiol.* 63 291–310. 10.1146/annurev.micro.091208.073435 19575560

[B58] Fisher M. C. Henk D. A. Briggs C. J. Brownstein J. S. Madoff L. C. McCraw S. L. (2012). Emerging fungal threats to animal, plant and ecosystem health. *Nature* 484 186–194. 10.1038/nature10947 22498624 PMC3821985

[B59] Forster S. C. Lawley T. D. (2015). Systematic discovery of probiotics. *Nat. Biotechnol.* 33 47–49. 10.1038/nbt.3111 25574637

[B60] Franzenburg S. Walter J. Künzel S. Wang J. Baines J. F. Bosch T. C. G. (2013). Distinct antimicrobial peptide expression determines host species-specific bacterial associations. *Proc. Natl. Acad. Sci. U.S.A.* 110 E3730–E3738. 10.1073/pnas.1304960110 24003149 PMC3785777

[B61] Franzosa E. A. Hsu T. Sirota-Madi A. Shafquat A. Abu-Ali G. Morgan X. C. (2015). Sequencing and beyond: integrating molecular “omics” for microbial community profiling. *Nat. Rev. Microbiol.* 13 360–372. 10.1038/nrmicro3451 25915636 PMC4800835

[B62] Fraune S. Anton-Erxleben F. Augustin R. Franzenburg S. Knop M. Schröder K. (2014). Bacteria–bacteria interactions within the microbiota of the ancestral metazoan Hydra contribute to fungal resistance. *ISME J.* 9 1543–1556. 10.1038/ismej.2014.239 25514534 PMC4478695

[B63] Freilich S. Kreimer A. Borenstein E. Yosef N. Sharan R. Gophna U. (2009). Metabolic-network-driven analysis of bacterial ecological strategies. *Genome Biol.* 10:R61 10.1186/gb-2009-10-6-r61 PMC2718495 19500338

[B64] Friedman J. Alm E. J. (2012). Inferring correlation networks from genomic survey data. *PLoS Comput. Biol.* 8:e1002687 10.1371/journal.pcbi.1002687 PMC3447976 23028285

[B65] Giannoukos G. Ciulla D. M. Huang K. Haas B. J. Izard J. Levin J. Z. (2012). Efficient and robust RNA-seq process for cultured bacteria and complex community transcriptomes. *Genome Biol.* 13:R23 10.1186/gb-2012-13-3-r23 PMC3439974 22455878

[B66] Gourbeyre P. Denery S. Bodinier M. (2011). Probiotics, prebiotics, and synbiotics: impact on the gut immune system and allergic reactions. *J. Leukoc. Biol.* 89 685–695. 10.1189/jlb.1109753 21233408

[B67] Greenblum S. Turnbaugh P. J. Borenstein E. (2012). Metagenomic systems biology of the human gut microbiome reveals topological shifts associated with obesity and inflammatory bowel disease. *Proc. Natl. Acad. Sci. U.S.A.* 109 594–599. 10.1073/pnas.1116053109 22184244 PMC3258644

[B68] Grice E. A. Segre J. A. (2011). The skin microbiome. *Nat. Rev. Microbiol.* 9 244–253. 10.1038/nrmicro2537 21407241 PMC3535073

[B69] Grzymski J. J. Murray A. E. Campbell B. J. Kaplarevic M. Gao G. R. Lee C. (2008). Metagenome analysis of an extreme microbial symbiosis reveals eurythermal adaptation and metabolic flexibility. *Proc. Natl. Acad. Sci. U.S.A.* 105 17516–17521. 10.1073/pnas.0802782105 18987310 PMC2579889

[B70] Gust K. A. Najar F. Z. Habib T. Lotufo G. R. Piggot A. M. Fouke B. W. (2014). Coral-zooxanthellae meta-transcriptomics reveals integrated response to pollutant stress. *BMC Genomics* 15:591 10.1186/1471-2164-15-591 PMC4117956 25016412

[B71] Harris R. N. Brucker R. M. Walke J. B. Becker M. H. Schwantes C. R. Flaherty D. C. (2009). Skin microbes on frogs prevent morbidity and mortality caused by a lethal skin fungus. *ISME J.* 3 818–824. 10.1038/ismej.2009.27 19322245

[B72] Harris R. N. James T. Y. Lauer A. Simon M. A. Patel A. (2006). Amphibian pathogen *Batrachochytrium dendrobatidis* is inhibited by the cutaneous bacteria of amphibian species. *Ecohealth* 3 53–56. 10.1007/s10393-005-0009-1

[B73] Hawkes C. V. Keitt T. H. (2015). Resilience vs. historical contingency in microbial responses to environmental change. *Ecol. Lett.* 18 612–625. 10.1111/ele.12451 25950733

[B74] Holden W. M. Hanlon S. M. Woodhams D. C. Chappell T. M. Wells H. L. Glisson S. M. (2015). Skin bacteria provide early protection for newly metamorphosed southern leopard frogs (*Rana sphenocephala*) against the frog-killing fungus, *Batrachochytrium dendrobatidis. Biol. Conserv.* 187 91–102. 10.1016/j.biocon.2015.04.007

[B75] Hoyt J. R. Cheng T. L. Langwig K. E. Hee M. M. Frick W. F. Kilpatrick A. M. (2015). Bacteria isolated from bats inhibit the growth of *Pseudogymnoascus destructans*, the causative agent of white-nose syndrome. *PLoS ONE* 10:e0121329 10.1371/journal.pone.0121329 PMC4390377 25853558

[B76] Huey R. B. (1991). Physiological consequences of habitat selection. *Am. Naturalist* 137 S91–S115. 10.1086/285141

[B77] Imangaliyev S. Keijser B. Crielaard W. Tsivtsivadze E. (2015). Personalized microbial network inference via co-regularized spectral clustering. *Methods* 83 28–35. 10.1016/j.ymeth.2015.03.017 25842007

[B78] Jani A. J. Briggs C. J. (2014). The pathogen *Batrachochytrium dendrobatidis* disturbs the frog skin microbiome during a natural epidemic and experimental infection. *Proc. Natl. Acad. Sci. U.S.A.* 111 E5049–E5058. 10.1073/pnas.1412752111 25385615 PMC4250152

[B79] Jorth P. Turner K. H. Gumus P. Nizam N. Buduneli N. Whiteley M. (2014). Metatranscriptomics of the human oral microbiome during health and disease. *MBio* 5:e01012-14. 10.1128/mBio.01012-14 PMC3977359 24692635

[B80] Katzenback B. A. Holden H. A. Falardeau J. Childers C. Hadj-Moussa H. Avis T. J. (2014). Regulation of the *Rana sylvatica* brevinin-1SY antimicrobial peptide during development and in dorsal and ventral skin in response to freezing, anoxia and dehydration. *J. Exp. Biol.* 217 1392–1401. 10.1242/jeb.092288 24436376

[B81] Kelsic E. D. Zhao J. Vetsigian K. Kishony R. (2015). Counteraction of antibiotic production and degradation stabilizes microbial communities. *Nature* 521 516–519. 10.1038/nature14485 25992546 PMC4551410

[B82] Kesarcodi-Watson A. Kaspar H. Lategan M. J. Gibson L. (2008). Probiotics in aquaculture: the need, principles and mechanisms of action and screening processes. *Aquaculture* 274 1–14. 10.1016/j.aquaculture.2007.11.019

[B83] Klemm P. Schembri M. A. (2000). Bacterial adhesins: function and structure. *Int. J. Med. Microbiol.* 290 27–35. 10.1016/S1438-4221(00)80102-2 11043979

[B84] Kline K. A. Fälker S. Dahlberg S. Normark S. Henriques-Normark B. (2009). Bacterial adhesins in host-microbe interactions. *Cell Host Microbe* 5 580–592. 10.1016/j.chom.2009.05.011 19527885

[B85] Knief C. Delmotte N. Chaffron S. Stark M. Innerebner G. Wassmann R. (2012). Metaproteogenomic analysis of microbial communities in the phyllosphere and rhizosphere of rice. *ISME J.* 6 1378–1390. 10.1038/ismej.2011.192 22189496 PMC3379629

[B86] Knights D. Costello E. K. Knight R. (2011a). Supervised classification of human microbiota. *FEMS Microbiol. Rev.* 35 343–359. 10.1111/j.1574-6976.2010.00251.x 21039646

[B87] Knights D. Kuczynski J. Koren O. Ley R. E. Field D. Knight R. (2011b). Supervised classification of microbiota mitigates mislabeling errors. *ISME J.* 5 570–573. 10.1038/ismej.2010.148 20927137 PMC3105748

[B88] Knights D. Parfrey L. W. Zaneveld J. Lozupone C. Knight R. (2011c). Human-associated microbial signatures: examining their predictive value. *Cell Host Microbe* 10 292–296. 10.1016/j.chom.2011.09.003 22018228 PMC3879110

[B89] Kolmogorov A. (1933). Sulla determinazione empirica di una legge di distribuzione. *G. Ital. Delgli Attuar* 4 83–91.

[B90] Kueneman J. G. Parfrey L. W. Woodhams D. C. Archer H. M. Knight R. McKenzie V. J. (2014). The amphibian skin-associated microbiome across species, space and life history stages. *Mol. Ecol.* 23 1238–1250. 10.1111/mec.12510 24171949

[B91] Kueneman J. G. Woodhams D. C. Van Treuren W. Archer H. M. Knight R. McKenzie V. J. (2015). Inhibitory bacteria reduce fungi on early life stages of endangered Colorado boreal toads (*Anaxyrus boreas*). *ISME J.* 10.1038/ismej.2015.168 [Epub ahead of print]. PMC4796932 26565725

[B92] Küng D. Bigler L. Davis L. R. Gratwicke B. Griffith E. Woodhams D. C. (2014). Stability of microbiota facilitated by host immune regulation: informing probiotic strategies to manage amphibian disease. *PLoS ONE* 9:e87101 10.1371/journal.pone.0087101 PMC3906108 24489847

[B93] Lakshmanan V. Selvaraj G. Bais H. P. (2014). Functional soil microbiome: belowground solutions to an aboveground problem. *Plant Physiol.* 166 689–700. 10.1104/pp.114.245811 25059708 PMC4213098

[B94] Langille M. G. I. Zaneveld J. Caporaso J. G. McDonald D. Knights D. Reyes J. A. (2013). Predictive functional profiling of microbial communities using 16S rRNA marker gene sequences. *Nat. Biotechnol.* 31 814–821. 10.1038/nbt.2676 23975157 PMC3819121

[B95] Langwig K. E. Voyles J. Wilber M. Q. Frick W. F. Murray K. A. Bolker B. M. (2015). Context-dependent conservation responses to emerging wildlife diseases. *Front. Ecol. Environ.* 13:195–202. 10.1890/140241

[B96] Lauer A. Simon M. Banning J. L. Andre E. Duncan K. Harris R. N. (2007). Common cutaneous bacteria from the eastern red-backed salamander can inhibit pathogenic fungi. *Copeia* 2007 630–640. 10.1643/0045-8511(2007)2007[630:CCBFTE]2.0.CO;2

[B97] Lawley T. D. Clare S. Walker A. W. Stares M. D. Connor T. R. Raisen C. (2012). Targeted restoration of the intestinal microbiota with a simple, defined bacteriotherapy resolves relapsing Clostridium difficile disease in mice. *PLoS Pathog* 8:e1002995 10.1371/journal.ppat.1002995 PMC3486913 23133377

[B98] Levy R. Borenstein E. (2012). Reverse ecology: from systems to environments and back. *Evol. Syst. Biol.* 751 329–353. 10.1007/978-1-4614-3567-9 22821465

[B99] Levy R. Borenstein E. (2013). Metabolic modeling of species interaction in the human microbiome elucidates community-level assembly rules. *Proc. Natl. Acad. Sci. U.S.A.* 110 12804–12809. 10.1073/pnas.1300926110 23858463 PMC3732988

[B100] Levy R. Borenstein E. (2014). Metagenomic systems biology and metabolic modeling of the human microbiome. From species composition to community assembly rules. *Gut Microbes* 5 1–6. 10.4161/gmic.28261 PMC4063856 24637600

[B101] Liao H.-L. Chen Y. Bruns T. D. Peay K. G. Taylor J. W. Branco S. (2014). Metatranscriptomic analysis of ectomycorrhizal roots reveals genes associated with *Piloderma*-*Pinus* symbiosis: improved methodologies for assessing gene expression in situ. *Environ. Microbiol.* 16 3730–3742. 10.1111/1462-2920.12619 25186788

[B102] Loftus S. C. House L. L. Hughey M. C. Walke J. B. Becker M. H. Belden L. K. (2015). *Dimension Reduction for Multinomial Models via a Kolmogorov-Smirnov Measure (KSM).* Blacksburg, VA: Department of Statistics, Virginia Tech.

[B103] Loman N. J. Pallen M. J. (2015). Twenty years of bacterial genome sequencing. *Nat. Rev. Microbiol.* 13 787–794. 10.1038/nrmicro3565 26548914

[B104] Loudon A. H. Woodhams D. C. Parfrey L. W. Archer H. Knight R. McKenzie V. (2014a). Microbial community dynamics and effect of environmental microbial reservoirs on red-backed salamanders (*Plethodon cinereus*). *ISME J.* 8 830–840. 10.1038/ismej.2013.200 24335825 PMC3960541

[B105] Loudon A. Holland J. Umile T. Burzynski E. Minbiole K. P. C. Harris R. N. (2014b). Interactions between amphibians’ symbiotic bacteria cause the production of emergent anti-fungal metabolites. *Front. Microbiol.* 5:441 10.3389/fmicb.2014.00441 PMC4139739 25191317

[B106] Love M. I. Huber W. Anders S. (2014). Moderated estimation of fold change and dispersion for RNA-Seq data with DESeq2. *Gen. Biol.* 15 1–21. 10.1101/002832 PMC4302049 25516281

[B107] Lovell D. M. W. Taylor J. Zwart A. Helliwell C. (2010). *Caution! Compositions! can Constraints on Omics Data Lead Analyses Astray?* CSIRO Report Number: EP10994. Dickson, ACT: CSIRO Available at: http://www.csiro.au/en/Organisation-Structure/Divisions/Computational-Informatics/Caution-Compositions.aspx

[B108] Macfoy C. Danosus D. Sandit R. Jones T. H. Garraffo H. M. Spande T. F. (2005). Alkaloids of anuran skin: antimicrobial function? *Z. Naturforsch. C* 60 932–937. 16402556 10.1515/znc-2005-11-1218

[B109] Manor O. Levy R. Borenstein E. (2014). Mapping the inner workings of the microbiome: genomic- and metagenomic-based study of metabolism and of metabolic interactions in the human gut microbiome. *Cell Metab.* 20 742–752. 10.1016/j.cmet.2014.07.021 25176148 PMC4252837

[B110] Martel A. Blooi M. Fisher M. C. Farrer R. A. Schmidt B. R. Tobler U. (2014). Recent introduction of a chytrid fungus endangers Western *Palearctic salamanders*. *Science* 346 630–631. 10.1126/science.1258268 25359973 PMC5769814

[B111] Martel A. Spitzen-van der Sluijs A. Blooi M. Bert W. Ducatelle R. Fisher M. C. (2013). *Batrachochytrium salamandrivorans* sp. nov. causes lethal chytridiomycosis in amphibians. *Proc. Natl. Acad. Sci. U.S.A.* 110 15325–15329. 10.1073/pnas.1307356110 24003137 PMC3780879

[B112] McGettigan P. A. (2013). Transcriptomics in the RNA-seq era. *Curr. Opin. Chem. Biol.* 17 4–11. 10.1016/j.cbpa.2012.12.008 23290152

[B113] McHardy I. H. Goudarzi M. Tong M. Ruegger P. M. Schwager E. Weger J. R. (2013). Integrative analysis of the microbiome and metabolome of the human intestinal mucosal surface reveals exquisite inter-relationships. *Microbiome* 1:17 10.1186/2049-2618-1-17 PMC3971612 24450808

[B114] McKenzie V. J. Bowers R. M. Fierer N. Knight R. Lauber C. L. (2012). Co-habiting amphibian species harbor unique skin bacterial communities in wild populations. *ISME J.* 6 588–596. 10.1038/ismej.2011.129 21955991 PMC3280140

[B115] McMahon T. A. Sears B. F. Venesky M. D. Bessler S. M. Brown J. M. Deutsch K. (2014). Amphibians acquire resistance to live and dead fungus overcoming fungal immunosuppression. *Nature* 511 224–227. 10.1038/nature13491 25008531 PMC4464781

[B116] McMurdie P. J. Holmes S. (2014). Waste not, want not: why rarefying microbiome data is inadmissible. *PLoS Comput. Biol.* 10:e1003531 10.1371/journal.pcbi.1003531 PMC3974642 24699258

[B117] Meng C. Kuster B. Culhane A. C. Moghaddas Gholami A. (2014). A multivariate approach to the integration of multi-omics datasets. *BMC Bioinformatics* 15:162 10.1186/1471-2105-15-162 PMC4053266 24884486

[B118] Metzker M. L. (2010). Sequencing technologies - the next generation. *Nat. Rev. Genet.* 11 31–46. 10.1038/nrg2626 19997069

[B119] Meyer E. A. Cramp R. L. Bernal M. H. Franklin C. E. (2012). Changes in cutaneous microbial abundance with sloughing: possible implications for infection and disease in amphibians. *Dis. Aquat. Organ.* 101 235–242. 10.3354/dao02523 23324420

[B120] Michaels C. J. Antwis R. E. Preziosi R. F. (2014). Impact of plant cover on fitness and behavioural traits of captive red-eyed tree frogs (*Agalychnis callidryas*). *PLoS ONE* 9:e95207 10.1371/journal.pone.0095207 PMC3989275 24740289

[B121] Muletz C. R. Myers J. M. Domangue R. J. Herrick J. B. Harris R. N. (2012). Soil bioaugmentation with amphibian cutaneous bacteria protects amphibian hosts from infection by *Batrachochytrium dendrobatidis*. *Biol. Conserv.* 152 119–126. 10.1016/j.biocon.2012.03.022

[B122] Myers J. M. Ramsey J. P. Blackman A. L. Nichols A. E. Minbiole K. P. C. Harris R. N. (2012). Synergistic inhibition of the lethal fungal pathogen *Batrachochytrium dendrobatidis*: the combined effect of symbiotic bacterial metabolites and antimicrobial peptides of the frog *Rana muscosa*. *J. Chem. Ecol.* 38 958–965. 10.1007/s10886-012-0170-2 22914957

[B123] Nakatsuji T. Chiang H.-I. Jiang S. B. Nagarajan H. Zengler K. Gallo R. L. (2013). The microbiome extends to subepidermal compartments of normal skin. *Nat. Commun.* 4:1431 10.1038/ncomms2441 PMC3655727 23385576

[B124] Ohmer M. E. B. Cramp R. L. White C. R. Franklin C. E. (2014). Skin sloughing rate increases with chytrid fungus infection load in a susceptible amphibian. *Funct. Ecol.* 29 674–682. 10.1111/1365-2435.12370

[B125] Onaka H. Mori Y. Igarashi Y. Furumai T. (2011). Mycolic acid-containing bacteria induce natural-product biosynthesis in *Streptomyces* species. *Appl. Environ. Microbiol.* 77 400–406. 10.1128/AEM.01337-10 21097597 PMC3020563

[B126] Papadimitriou K. Zoumpopoulou G. Foligné B. Alexandraki V. Kazou M. Pot B. (2015). Discovering probiotic microorganisms: in vitro, in vivo, genetic and omics approaches. *Front. Microbiol.* 6:58 10.3389/fmicb.2015.00058 PMC4330916 25741323

[B127] Parfrey L. W. Walters W. A. Lauber C. L. Clemente J. C. Berg-Lyons D. Teiling C. (2014). Communities of microbial eukaryotes in the mammalian gut within the context of environmental eukaryotic diversity. *Front. Microbiol.* 5:298 10.3389/fmicb.2014.00298 PMC4063188 24995004

[B128] Pask J. D. Woodhams D. C. Rollins-Smith L. A. (2012). The ebb and flow of antimicrobial skin peptides defends northern leopard frogs (*Rana pipiens*) against chytridiomycosis. *Glob. Change Biol.* 18 1231–1238. 10.1111/j.1365-2486.2011.02622.x

[B129] Patel R. M. Denning P. W. (2013). Therapeutic use of prebiotics, probiotics, and postbiotics to prevent necrotizing enterocolitis: what is the current evidence? *Clin. Perinatol.* 40 11–25. 10.1016/j.clp.2012.12.002 23415261 PMC3575601

[B130] Patti G. J. Tautenhahn R. Rinehart D. Cho K. Shriver L. P. Manchester M. (2013). A view from above: cloud plots to visualize global metabolomic data. *Anal. Chem.* 85 798–804. 10.1021/ac3029745 23206250 PMC3716252

[B131] Pearson K. (1909). Determination of the coefficient of correlation. *Science* 30 23–25. 10.1126/science.30.757.23 17838275

[B132] Piotrowski J. S. Annis S. L. Longcore J. E. (2004). Physiology of *Batrachochytrium dendrobatidis*, a chytrid pathogen of amphibians. *Mycologia* 96 9–15. 10.2307/3761981 21148822

[B133] Price S. J. Garner T. W. J. Balloux F. Ruis C. Paszkiewicz K. H. Moore K. (2015). A de novo assembly of the common frog (*Rana temporaria*) transcriptome and comparison of transcription following exposure to *Ranavirus* and *Batrachochytrium dendrobatidis*. *PLoS ONE* 10:e0130500 10.1371/journal.pone.0130500 PMC4481470 26111016

[B134] Price S. J. Garner T. W. J. Nichols R. A. Balloux F. Ayres C. Mora-Cabello de Alba A. (2014). Collapse of amphibian communities due to an introduced *Ranavirus*. *Curr. Biol.* 24 2586–2591. 10.1016/j.cub.2014.09.028 25438946

[B135] R Core Team (2014). *R: A Language and Environment for Statistical Computing.* Vienna: R Foundation for Statistical Computing. Available at: http://www.R-project.org/

[B136] Raffel T. R. Rohr J. R. Kiesecker J. M. Hudson P. J. (2006). Negative effects of changing temperature on amphibian immunity under field conditions. *Funct. Ecol.* 20 819–828. 10.1111/j.1365-2435.2006.01159.x

[B137] Ramsey J. P. Reinert L. K. Harper L. K. Woodhams D. C. Rollins-Smith L. A. (2010). Immune defenses against *Batrachochytrium dendrobatidis*, a fungus linked to global amphibian declines, in the South African clawed frog, Xenopus laevis. *Infect. Immun.* 78 3981–3992. 10.1128/IAI.00402-10 20584973 PMC2937463

[B138] Rappé M. S. Connon S. A. Vergin K. L. Giovannoni S. J. (2002). Cultivation of the ubiquitous SAR11 marine bacterioplankton clade. *Nature* 418 630–633. 10.1038/nature00917 12167859

[B139] Rebollar E. A. Hughey M. C. Medina D. Harris R. N. Ibáñez R. Belden L. K. (2016). Skin bacterial diversity of Panamanian frogs is associated with host susceptibility and presence of *Batrachochytrium dendrobatidis*. *ISME J.* 10.1038/ismej.2015.234 [Epub ahead of print]. PMC4918441 26744810

[B140] Reid G. Younes J. A. Van der Mei H. C. Gloor G. B. Knight R. Busscher H. J. (2011). Microbiota restoration: natural and supplemented recovery of human microbial communities. *Nat. Rev. Microbiol.* 9 27–38. 10.1038/nrmicro2473 21113182

[B141] Reshef D. N. Reshef Y. A. Finucane H. K. Grossman S. R. Mcvean G. Turnbaugh P. J. (2011). Detecting novel associations in large data sets. *Science* 334 1518–1524. 10.1126/science.1205438 22174245 PMC3325791

[B142] Ringø E. Olsen R. E. Gifstad T. Dalmo R. A. Amlund H. Hemre G. I. (2010). Prebiotics in aquaculture: a review. *Aquac. Nutr.* 16 117–136. 10.1111/j.1365-2095.2009.00731.x

[B143] Rizzetto L. Filippo C. De Cavalieri D. (2015). Fungal genomics & biology mycobiota: micro-eukaryotes inhabiting our body as commensals or opportunistic pathogens. *Fungal Genom. Biol.* 4 1–9. 10.4172/2165-8056.1000120

[B144] Roberts D. W. (2007). *LaBdsv: Ordination and Multivariate Analysis for Ecology. R Package Version, 1.3-1.* Available at: http://CRAN.R-project.org/package=labdsv

[B145] Rollins-Smith L. A. Ramsey J. P. Pask J. D. Reinert L. K. Woodhams D. C. (2011). Amphibian immune defenses against chytridiomycosis: impacts of changing environments. *Integr. Comp. Biol.* 51 552–562. 10.1093/icb/icr095 21816807

[B146] Rollins-Smith L. A. Woodhams D. C. (2012). “Amphibian immunity: staying in tune with the environment,” in *Eco-Immunology*, eds Demas G. E. Nelson R. J. (Oxford: Oxford University Press), 92–143.

[B147] Rosvall M. Bergstrom C. T. (2010). Mapping change in large networks. *PLoS ONE* 5:e8694 10.1371/journal.pone.0008694 PMC2811724 20111700

[B148] Rowley J. J. L. Alford R. A. (2013). Hot bodies protect amphibians against chytrid infection in nature. *Sci. Rep.* 3:1515 10.1038/srep01515 PMC3604863 23519020

[B149] Ruan Q. Dutta D. Schwalbach M. S. Steele J. A. Fuhrman J. A. Sun F. (2006). Local similarity analysis reveals unique associations among marine bacterioplankton species and environmental factors. *Bioinformatics* 22 2532–2538. 10.1093/bioinformatics/btl417 16882654

[B150] Sánchez B. Ruiz L. Gueimonde M. Margolles A. (2013). Omics for the study of probiotic microorganisms. *Food Res. Int.* 54 1061–1071. 10.1016/j.foodres.2013.01.029

[B151] Sanders J. G. Powell S. Kronauer D. J. C. Vasconcelos H. L. Frederickson M. E. Pierce N. E. (2014). Stability and phylogenetic correlation in gut microbiota: lessons from ants and apes. *Mol. Ecol.* 23 1268–1283. 10.1111/mec.12611 24304129

[B152] Savage A. E. Kiemnec-Tyburczy K. M. Ellison A. R. Fleischer R. C. Zamudio K. R. (2014). Conservation and divergence in the frog immunome: pyrosequencing and de novo assembly of immune tissue transcriptomes. *Gene* 542 98–108. 10.1016/j.gene.2014.03.051 24680726

[B153] Savage A. E. Zamudio K. R. (2011). MHC genotypes associate with resistance to a frog-killing fungus. *Proc. Natl. Acad. Sci. U.S.A.* 108 16705–16710. 10.1073/pnas.1106893108 21949385 PMC3189034

[B154] Schloegel L. M. Toledo L. F. Longcore J. E. Greenspan S. E. Vieira C. A. Lee M. (2012). Novel, panzootic and hybrid genotypes of amphibian chytridiomycosis associated with the bullfrog trade. *Mol. Ecol.* 21 5162–5177. 10.1111/j.1365-294X.2012.05710.x 22857789

[B155] Schloss P. D. Allen H. K. Klimowicz A. K. Mlot C. Gross J. A. Savengsuksa S. (2010). Psychrotrophic strain of *Janthinobacterium lividum* from a cold Alaskan soil produces prodigiosin. *DNA Cell Biol.* 29 533–541. 10.1089/dna.2010.1020 20626288 PMC6468942

[B156] Schrope M. (2014). Sea star wasting. *Proc. Natl. Acad. Sci. U.S.A.* 111:6855 10.1073/pnas.1404650111 PMC4024922 24825882

[B157] Schulze S. Henkel S. G., Driesch D. Guthke R. Linde J. (2015). Computational prediction of molecular pathogen-host interactions based on dual transcriptome data. *Front. Microbiol.* 6:65 10.3389/fmicb.2015.00065 PMC4319478 25705211

[B158] Searle C. L. Gervasi S. S. Hua J. Hammond J. I. Relyea R. A. Olson D. H. (2011). Differential host susceptibility to *Batrachochytrium dendrobatidis*, an emerging amphibian pathogen. *Conserv. Biol.* 25 965–974. 10.1111/j.1523-1739.2011.01708.x 21732979

[B159] Segata N. Izard J. Waldron L. Gevers D. Miropolsky L. Garrett W. S. (2011). Metagenomic biomarker discovery and explanation. *Genome Biol.* 12:R60 10.1186/gb-2011-12-6-r60 PMC3218848 21702898

[B160] Shannon P. Markiel A. Ozier O. Baliga N. S. Wang J. T. Ramage D. (2003). Cytoscape: a software environment for integrated models of biomolecular interaction networks. *Genome Res.* 13 2498–2504. 10.1101/gr.1239303 14597658 PMC403769

[B161] Sharaf M. A. Illman D. L. Kowalski B. R. (eds) (1986). *Chemometrics.* New York, NY: Wiley.

[B162] Smirnov N. (1936). Sur la distribution de ω2 (criterium de M. R. v. Mises). *Compt. Rend. Acad. Sci.* 202 449–452.

[B163] Spearman C. (1904). The proof and measurement of association between two things. *Am. J. Psychol.* 15 72–101. 10.2307/1412159 3322052

[B164] Spor A. Koren O. Ley R. (2011). Unravelling the effects of the environment and host genotype on the gut microbiome. *Nat. Rev. Microbiol.* 9 279–290. 10.1038/nrmicro2540 21407244

[B165] Statnikov A. Henaff M. Narendra V. Konganti K. Li Z. Yang L. (2013). A comprehensive evaluation of multicategory classification methods for microbiomic data. *Microbiome* 1:11 10.1186/2049-2618-1-11 PMC3960509 24456583

[B166] Steele J. A. Countway P. D. Xia L. Vigil P. D. Beman J. M. Kim D. Y. (2011). Marine bacterial, archaeal and protistan association networks reveal ecological linkages. *ISME J.* 5 1414–1425. 10.1038/ismej.2011.24 21430787 PMC3160682

[B167] Stevenson L. A. Alford R. A. Bell S. C. Roznik E. A. Berger L. Pike D. A. (2013). Variation in thermal performance of a widespread pathogen, the amphibian chytrid fungus *Batrachochytrium dendrobatidis*. *PLoS ONE* 8:e7380 10.1371/journal.pone.0073830 PMC3762749 24023908

[B168] Stewart F. J. Sharma A. K. Bryant J. A. Eppley J. M. DeLong E. F. (2011). Community transcriptomics reveals universal patterns of protein sequence conservation in natural microbial communities. *Genome Biol.* 12:R26 10.1186/gb-2011-12-3-r26 PMC3129676 21426537

[B169] Stice M. J. Briggs C. J. (2010). Immunization is ineffective at preventing infection and mortality due to the amphibian chytrid fungus *Batrachochytrium dendrobatidis*. *J. Wildl. Dis.* 46 70–77. 10.7589/0090-3558-46.1.70 20090019

[B170] Susi H. Barrès B. Vale P. F. Laine A.-L. (2015). Co-infection alters population dynamics of infectious disease. *Nat. Commun.* 6:5975 10.1038/ncomms6975 PMC4354079 25569306

[B171] Tennessen J. A. Blouin M. S. (2007). Selection for antimicrobial peptide diversity in frogs leads to gene duplication and low allelic variation. *J. Mol. Evol.* 65 605–615. 10.1007/s00239-007-9045-5 17938991

[B172] Tennessen J. A. Woodhams D. C. Chaurand P. Reinert L. K. Billheimer D. Shyr Y. (2009). Variations in the expressed antimicrobial peptide repertoire of northern leopard frog (*Rana pipiens*) populations suggest intraspecies differences in resistance to pathogens. *Dev. Comp. Immunol.* 33 1247–1257. 10.1016/j.dci.2009.07.004 19622371 PMC2927990

[B173] Umile T. P. McLaughlin P. J. Johnson K. R. Honarvar S. Blackman A. L. Burzynski E. A. (2014). Nonlethal amphibian skin swabbing of cutaneous natural products for HPLC fingerprinting. *Anal. Methods* 6 3277–3284. 10.1039/c4ay00566j

[B174] Underhill D. M. Iliev I. D. (2014). The mycobiota: interactions between commensal fungi and the host immune system. *Nat. Rev. Immunol.* 14 405–416. 10.1038/nri3684 24854590 PMC4332855

[B175] Uyeno Y. Shigemori S. Shimosato T. (2015). Effect of probiotics/prebiotics on cattle health and productivity. *Microbes Environ.* 30 126–132. 10.1264/jsme2.ME14176 26004794 PMC4462921

[B176] Vartoukian S. R. Palmer R. M. Wade W. G. (2010). Strategies for culture of “unculturable” bacteria. *FEMS Microbiol. Lett.* 309 1–7. 10.1111/j.1574-6968.2010.02000.x 20487025

[B177] Voyles J. Johnson L. R. Briggs C. J. Cashins S. D. Alford R. A. Berger L. (2012). Temperature alters reproductive life history patterns in *Batrachochytrium dendrobatidis*, a lethal pathogen associated with the global loss of amphibians. *Ecol. Evol.* 2 2241–2249. 10.1002/ece3.334 23139882 PMC3488674

[B178] Wake D. B. Vredenburg V. T. (2008). Are we in the midst of the sixth mass extinction? A view from the world of amphibians. *Proc. Natl. Acad. Sci. U.S.A.* 105 11466–11473. 10.1073/pnas.0801921105 18695221 PMC2556420

[B179] Walke J. B. Becker M. H. Hughey M. C. Swartwout M. C. Jensen R. V. Belden L. K. (2015). Most of the dominant members of amphibian skin bacterial communities can be readily cultured. *Appl. Environ. Microbiol.* 81 6589–6600. 10.1128/AEM.01486-15 26162880 PMC4561701

[B180] Walke J. B. Becker M. H. Loftus S. C. House L. L. Cormier G. Jensen R. V. (2014). Amphibian skin may select for rare environmental microbes. *ISME J.* 8 1–11. 10.1038/ismej.2014.77 24858782 PMC4992085

[B181] Walke J. B. Harris R. N. Reinert L. K. Rollins-Smith L. A. Woodhams D. C. (2011). Social immunity in amphibians: evidence for vertical transmission of innate defenses. *Biotropica* 43 396–400. 10.1111/j.1744-7429.2011.00787.x

[B182] Watrous J. Roach P. Alexandrov T. Heath B. S. Yang J. Y. Kersten R. D. (2012). Mass spectral molecular networking of living microbial colonies. *Proc. Natl. Acad. Sci. U.S.A.* 109 E1743–E1752. 10.1073/pnas.1203689109 22586093 PMC3387089

[B183] Watve M. Shejval V. Sonawane C. Rahalkar M. Matapurkar A. Shouche Y. (2000). The “K” selected oligotrhophic bacteria: a key to uncultured diversity? *Curr. Sci.* 78 1535–1542.

[B184] Weiss S. J. Xu Z. Amir A. Peddada S. Bittinger K. Gonzalez A. (2015). Effects of library size variance, sparsity, and compositionality on the analysis of microbiome data. *Peer J.* 3:e1408 10.7287/peerj.preprints.1157v1

[B185] Westermann A. J. Gorski S. A. Vogel J. (2012). Dual RNA-seq of pathogen and host. *Nat. Rev. Microbiol.* 10 618–630. 10.1038/nrmicro2852 22890146

[B186] Wiggins P. J. Smith J. M. Harris R. N. Minbiole K. P. C. (2011). The gut of red-backed salamanders (*Plethodon cinereus*) may serve as a reservoir for an antifungal cutaneous bacterium. *J. Herpetol.* 45 329–332. 10.1670/10-231.1

[B187] Wishart D. S. Jewison T. Guo A. C. Wilson M. Knox C. Liu Y. (2013). HMDB 3.0-the human metabolome database in 2013. *Nucleic Acids Res.* 41 801–807. 10.1093/nar/gks1065 PMC3531200 23161693

[B188] Wishart D. S. Knox C. Guo A. C. Eisner R. Young N. Gautam B. (2009). HMDB: a knowledgebase for the human metabolome. *Nucleic Acids Res.* 37 603–610. 10.1093/nar/gkn810 PMC2686599 18953024

[B189] Wishart D. S. Tzur D. Knox C. Eisner R. Guo A. C. Young N. (2007). HMDB: the human metabolome database. *Nucleic Acids Res.* 35 521–526. 10.1093/nar/gkl923 PMC1899095 17202168

[B190] Woodhams D. Alford R. A. Briggs C. J. (2008). Life-history trade-offs influence disease in changing climates: strategies of an amphibian pathogen. *Ecology* 89 1627–1639. 10.1890/06-1842.1 18589527

[B191] Woodhams D. C. Alford R. A. Antwis R. E. Archer H. Becker M. H. Belden L. K. (2015). Antifungal isolates database of amphibian skin-associated bacteria and function against emerging fungal pathogens. *Ecology* 96:595 10.1890/14-1837.1

[B192] Woodhams D. C. Bigler L. Marschang R. (2012). Tolerance of fungal infection in European water frogs exposed to *Batrachochytrium dendrobatidis* after experimental reduction of innate immune defenses. *BMC Vet. Res.* 8:197 10.1186/1746-6148-8-197 PMC3485127 23088169

[B193] Woodhams D. C. Brandt H. Baumgartner S. Kielgast J. Küpfer E. Tobler U. (2014). Interacting symbionts and immunity in the amphibian skin mucosome predict disease risk and probiotic effectiveness. *PLoS ONE* 9:e96375 10.1371/journal.pone.0096375 PMC4005770 24789229

[B194] Woodhams D. C. Vredenburg V. T. Simon M.-A. Billheimer D. Shakhtour B. Shyr Y. (2007). Symbiotic bacteria contribute to innate immune defenses of the threatened mountain yellow-legged frog, *Rana muscosa. Biol. Conserv.* 138 390–398. 10.1016/j.biocon.2007.05.004

[B195] Woyke T. Teeling H. Ivanova N. N. Huntemann M. Richter M. Gloeckner F. O. (2006). Symbiosis insights through metagenomic analysis of a microbial consortium. *Nature* 443 950–955. 10.1038/nature05192 16980956

[B196] Xia L. C. Ai D. Cram J. Fuhrman J. A. Sun F. (2013). Efficient statistical significance approximation for local similarity analysis of high-throughput time series data. *Bioinformatics* 29 230–237. 10.1093/bioinformatics/bts668 23178636 PMC4990825

[B197] Xu Z. Hansen M. A. Hansen L. H. Jacquiod S. Sørensen S. J. (2014). Bioinformatic approaches reveal metagenomic characterization of soil microbial community. *PLoS ONE* 9:e93445 10.1371/journal.pone.0093445 PMC3972102 24691166

[B198] Yap B. T. A. Koo M. S. Ambrose R. F. Wake D. B. Vredenburg V. T. (2015). Averting a North American biodiversity crisis. *Science* 349 481–482. 10.1126/science.aab1052 26228132

[B199] Zeng B. Han S. Wang P. Wen B. Jian W. Guo W. (2015). The bacterial communities associated with fecal types and body weight of rex rabbits. *Sci. Rep.* 5:9342 10.1038/srep09342 PMC4366860 25791609

[B200] Zengler K. Toledo G. Rappe M. Elkins J. Mathur E. J. Short J. M. (2002). Cultivating the uncultured. *Proc. Natl. Acad. Sci. U.S.A.* 99 15681–15686. 10.1073/pnas.252630999 12438682 PMC137776

[B201] Zhou J. Deng Y. Luo F. He Z. Yang Y. (2011). Phylogenetic molecular ecological network of soil microbial communities in response to elevated CO_2_. *MBio* 2:e122-11. 10.1128/mBio.00122-11 PMC3143843 21791581

